# Membrane-assisted tariquidar access and binding mechanisms of human ATP-binding cassette transporter P-glycoprotein

**DOI:** 10.3389/fmolb.2024.1364494

**Published:** 2024-03-15

**Authors:** Yingjie Gao, Caiyan Wei, Lanxin Luo, Yang Tang, Yongzhen Yu, Yaling Li, Juan Xing, Xianchao Pan

**Affiliations:** ^1^ Department of Medicinal Chemistry, School of Pharmacy, Southwest Medical University, Luzhou, Sichuan, China; ^2^ Department of Pathophysiology, School of Basic Medical Science, Southwest Medical University, Luzhou, Sichuan, China; ^3^ Department of Pharmacy, The Affiliated Hospital, Southwest Medical University, Luzhou, Sichuan, China

**Keywords:** human P-glycoprotein, tariquidar, third-generation inhibitors, molecular dynamics simulations, mechanism of action, membrane lipids

## Abstract

The human multidrug transporter P-glycoprotein (P-gp) is physiologically essential and of key relevance to biomedicine. Recent structural studies have shed light on the mode of inhibition of the third-generation inhibitors for human P-gp, but the molecular mechanism by which these inhibitors enter the transmembrane sites remains poorly understood. In this study, we utilized all-atom molecular dynamics (MD) simulations to characterize human P-gp dynamics under a potent inhibitor, tariquidar, bound condition, as well as the atomic-level binding pathways in an explicit membrane/water environment. Extensive unbiased simulations show that human P-gp remains relatively stable in tariquidar-free and bound states, while exhibiting a high dynamic binding mode at either the drug-binding pocket or the regulatory site. Free energy estimations by partial nudged elastic band (PNEB) simulations and Molecular Mechanics Generalized Born Surface Area (MM/GBSA) method identify two energetically favorable binding pathways originating from the cytoplasmic gate with an extended tariquidar conformation. Interestingly, free tariquidar in the lipid membrane predominantly adopts extended conformations similar to those observed at the regulatory site. These results suggest that membrane lipids may preconfigure tariquidar into an active ligand conformation for efficient binding to the regulatory site. However, due to its conformational plasticity, tariquidar ultimately moves toward the drug-binding pocket in both pathways, explaining how it acts as a substrate at low concentrations. Our molecular findings propose a membrane-assisted mechanism for the access and binding of the third-generation inhibitors to the binding sites of human P-gp, and offer deeper insights into the molecule design of more potent inhibitors against P-gp-mediated drug resistance.

## 1 Introduction

P-glycoprotein (P-gp), also known as ABCB1 or MDR1, is a prominent member of ATP-binding cassette (ABC) family that mediates the translocation of diverse molecules across biological membranes in an ATP-dependent manner (Schinkel and Jonker, 2003). It is constitutively expressed in various human tissues, including the small intestine, liver, kidney, pancreas, as well as the blood-brain barrier (BBB), where it serves a physiological protective or excretory role against xenobiotics and endobiotics (Thiebaut et al., 1987; Fromm, 2004). As a consequence of broad substrate specificity, P-gp can transport a wide range of drugs out of the cells, thus greatly affecting their pharmacokinetic parameters, bioavailability and desired effects (Szakacs et al., 2008). Furthermore, overexpression of P-gp is often associated with multidrug resistance (MDR) in cancer cells, a major obstacle to effective treatment with many chemotherapeutic agents such as paclitaxel and vincristine, which are well characterized P-gp substrates (Gottesman et al., 2002; Eckford and Sharom, 2009; Robey et al., 2018). Therefore, targeted inhibition of site-specific P-gp is a promising strategy to improve the absorption for orally administered drugs, enhance the BBB penetrance of central nervous system (CNS) therapeutics, and reverse the MDR in cancer (Szakacs et al., 2006; Giacomini et al., 2010).

Due to the vital role in drug resistance and pharmacology, the structure and mechanism of P-gp have been intensely investigated. At the structural level, P-gp is characterized by a canonical ABC architecture, consisting of two transmembrane domains (TMDs) that recognize and transport substrates by alternating between inward-open and outward-open states, and two cytoplasmic nucleotide-binding domains (NBDs) that bind and hydrolyze ATP to drive the large-scale conformational changes necessary for substrate efflux (Kim and Chen, 2018). Recently, atomic resolution structures of P-gp from different species have been determined by X-ray crystallography and single-particle cryoelectron microscopy (cryo-EM), which provide important insights into the mode of action of substrates and inhibitors as well as the large-scale conformational changes during the transport cycle (Srikant and Gaudet, 2019). Specially, cryo-EM structures of inhibitor-bound human P-gp have revealed the inhibition mode in which the third-generation inhibitors (tariquidar or elacridar) are bound in pairs, simultaneously occupying the central drug-binding pocket and the access tunnel that is thought of as a regulatory site. However, the molecular mechanism of how these inhibitors access and bind to the transmembrane sites remains largely unknown, which hinders rational design and lead optimization. Previous studies have suggested that amphiphilic and lipophilic molecules may gain access to the binding sites by first partitioning into the membrane and then reaching the binding site via a lateral diffusion route through the proposed intramembranous entrance gates (Jin et al., 2012; Wen et al., 2013; Ferreira et al., 2015; Xu et al., 2015). The higher drug sensitivity of P-gp in the membrane than in detergents also suggests a membrane-mediated transporter access and binding mechanism (Romsicki and Sharom, 1999; Jin et al., 2012). Not surprisingly, the lipid membrane plays a critical role in modulating the drug interactions with P-gp, but the detailed molecular mechanism also remains elusive.

A close-up view of the dynamic binding process is exceedingly favored to extend our understanding of the drug-P-gp interactions. Current experimental techniques cannot monitor the process of a drug entry into the binding sites and the associated conformational changes at the atomic level. Alternatively, computational simulation techniques, such as molecular dynamics (MD) simulations, serve as a computational microscope to visit such dynamic processes with a high temporal and spatial resolution (Liu et al., 2016; Furuta, 2021). For instance, all-atom MD simulations were conducted to investigate the structural dynamics of mouse P-gp in the inward-open state. The simulations revealed that both apo (nucleotide-free) and ATP-bound forms of P-gp adopted a surprisingly wide range of conformations, as evidenced by the degree of separation between NBDs (Wen et al., 2013). To gain insight into the inhibition mechanism, Tajkhorshid and colleagues explored the conformational changes of mouse P-gp in the presence of tariquidar using all-atom simulations (Kapoor et al., 2021). The simulations revealed that the lipids penetrating into the P-gp cavity through the proposed entry portals enhanced the inhibitor-mediated conformational restriction, suggesting a synergistic role of lipids in modulating the conformational dynamics of P-gp and its transport inhibition (Kapoor et al., 2021). Moreover, many improved MD techniques, such as steered MD (Ferreira et al., 2015), partial nudged elastic band (PNEB) (Xing et al., 2019), random accelerated MD (Zhang et al., 2015), and umbrella sampling simulations (Subramanian et al., 2015; Subramanian et al., 2019) have been applied to characterize the dynamic process of ligand binding to the P-gp cavity. Recently, the transport pathway for the physiological substrate, uric acid of human ABCG2, another MDR ABC transporter, have been explored by biased MD simulations (Nagy et al., 2021). Additionally, extensive microsecond-scale MD simulations of the heterodimeric ABC transporter TM287/288, have captured an occluded intermediate in the large-scale conformational transitions between the inward- and outward-open states upon ATP binding (Goddeke et al., 2018). Subsequent simulations in the presence of daunorubicin or verapamil provided a detailed atomistic picture of the dynamic translocation pathways in TM287/288 (Goddeke and Schafer, 2020).

In this study, we employed MD simulations to further investigate the conformational dynamics of human P-gp at the level of the individual tariquidar molecule, as well as the molecular mechanisms of the inhibitor entry into the transmembrane sites. A set of unbiased simulations demonstrated that the cryo-EM structures of the human P-gp, especially the doubly occluded TMDs, are relatively stable, where the proposed entrance gates for drug entry into the transmembrane cavity remain in a semi-open state. However, the single tariquidar molecule bound in either the central pocket or the access tunnel shows positional changes and flexible binding. Microsecond-scale simulations of free tariquidar in aqueous solutions capture a dynamic equilibrium between the extended and folded conformations, suggesting high structural flexibility. Interestingly, the dynamic equilibrium shifts toward predominantly extended conformations in the lipid membrane. Following PNEB simulations reveal two energetically-favorable binding pathways for tariquidar, both of which are initiated from the cytoplasmic gate region with an extended conformation. Notably, the structural flexibility of tariquidar highly contributes to the binding to the central pocket rather than the regulatory site, suggesting that conformational restriction of the ligands may enhance the inhibitory activity and reduce the transport potential. According to these molecular findings, we suggest a novel binding mechanism for tariquidar, and potentially for the third-generation inhibitors, which highlights the role of membrane lipids in pre-organizing the ligand in an active conformation for the access and binding to the transmembrane sites in human P-gp. Our results also provide important implications for the molecular design of more potent and selective inhibitors targeting human P-gp.

## 2 Materials and methods

### 2.1 Structural preparation

The starting structures for all-atom MD simulations were taken from the cryo-EM structures of the drug-free (apo) (PDB ID: 7A65) and the doubly inhibitor-bound P-gp (PDB ID: 7A6E) adopting an inward-occluded conformation with two tariquidar molecules bound in the central pocket and the access tunnel site, respectively (Nosol et al., 2020). The singly tariquidar-bound structures were generated by removing one tariquidar molecule bound in the transmembrane sites. Additional apo structure for control simulations was obtained by removing the two tariquidar molecules. In all structural models, the monoclonal antibody MRK16 and cholesterol molecules used for structural determination were removed. The missing extracellular loop (residues 86–103) connecting TM1 to TM2 was modeled using Modeller (Webb and Sali, 2016), and the mutation A893S was reverted to the wild-type sequence. The highly flexible linker region (residues 631–693) connecting NBD1 to TMD2 was missing in all the currently available structures of P-gp and was not modeled to reduce the risk of bias in this study. Furthermore, all the cryo-EM structures were determined in the absence of nucleotides, and therefore any nucleotides were not added in the ATP-binding sites within NBDs. The protonation states of titratable residues were assigned by pKa predictions at pH 7.0 using PROPKA (Olsson et al., 2011). The N- and C-terminal residues of the protein were modeled as neutral. Additionally, the extended and folded tariquidar poses were extracted from the cryo-EM structure of doubly tariquidar-bound P-gp (PDB ID: 7A6E) to simulate its conformational behavior in different environments.

### 2.2 Simulation systems

The above-obtained structures were used to build the simulation systems in an explicit membrane/water environment using CHARMM-GUI (Jo et al., 2008). The orientation of the protein in the bilayer was determined with the OPM (Orientations of Proteins in Membranes) web server (Lomize et al., 2012). The structures were inserted into a lipid bilayer composed of 335 1-palmitoyl-2-oleoyl-sn-glycero-3-phosphocholine (POPC) lipids (120 lipids in the upper leaflet, 115 in the lower leaflet) using the CHARMM-GUI membrane builder module (Jo et al., 2009). The protein/membrane complex was solvated with TIP3P water molecules padding on both sides of the bilayer, and then neutralized with a 0.15 M concentration of NaCl, yielding a total system size of ca. 169,500 atoms with a box dimension of ca. 10.0 × 10.0 × 18.9 nm. To simulate the behavior of tariquidar in water solution and membrane environments, a single tariquidar molecule was separately placed in a cubic TIP3P water box with a padding of 1.0 nm from the edge of the box to any compound atom or in the hydrophobic core of a hydrated POPC bilayer consisting of 36 lipids per leaflet. To enhance conformational sampling, both of the extended and folded poses of tariquidar were selected as starting structures. The system charge was also neutralized with a 0.15 M concentration of NaCl. The final systems contained ca. 7,800 and 17,300 atoms for tariquidar simulations in the water and membrane, respectively.

### 2.3 Unbiased MD protocol

Unbiased MD simulations were carried out using AMBER18 program package (Salomon-Ferrer et al., 2013). The AMBER ff19SB force field was used for the protein (Tian et al., 2020), together with the LIPID17 parameters for POPC, allowing for tensionless lipid bilayer simulations in AMBER. Tariquidar was parameterized with the general AMBER force field, version 2 (GAFF2) that is recommended to be compatible with the AMBER ff19SB protein force field used (He et al., 2020). The atomic partial charges were derived using AM1-BCC model (Jakalian et al., 2002). Prior to the production simulations, all the systems were subjected to 10,000 steps of energy minimization (5,000 steps of the steepest descent and 5,000 steps of conjugated gradient minimization), and then equilibrated for 10 ns according to the CHARMM-GUI six-step equilibration protocol. Briefly, the system was equilibrated in an NVT ensemble for the first two equilibration steps followed by an NPT ensemble for the last four steps at a constant temperature of 310 K. In the equilibration protocol, harmonic position restraints were applied on the protein-ligand complex and the headgroups of the lipids with a gradually decreased force constant from 10.0 to 0.1 kcal/mol/Å^2^, allowing for relaxation of the lipid and water molecules around the protein. Additional dihedral restraints were used to restrain the chirality of lipid headgroups and double bonds by a force constant of 5.0 kcal/mol/Å^2^ that was slowly released in the equilibration process.

Following production simulations were performed under periodic boundary conditions in the NPT ensemble without any restraints. An integration time step of 2 fs was used for all unbiased simulations. The semi-isotropic Berendsen coupling barostat with compressibility of 4.5 × 10^−5^ bar^−1^ was used to maintain the pressure at 1 bar by separately coupling the lateral (xy) and normal (z) box directions, and the Langevin thermostat was used to maintain the temperature at 310 K with a collision frequency of 1.0 ps^−1^. The SETTLE algorithm was used to constrain the internal degrees of freedom of the water molecules, and the SHAKE algorithm was used to constrain all bonds involving hydrogen atoms (Ryckaert et al., 1977). Long-range electrostatic interactions were calculated using the partial mesh Ewald (PME) method with an interpolation order of 6 (Darden et al., 1993). A nonbonded cutoff of 10 Å was used for the van der Waals and short-range electrostatic interactions. For each of the P-gp systems, two independent MD simulations, each for 500 ns, were initiated from equilibrated structures with different random seeds for the initial velocity assignment. For the single tariquidar systems (i.e., without P-gp), three independent simulations, each of length 500 ns, were initiated from the folded and extended conformations, yielding a total of 3 μs unbiased sampling of tariquidar in the water solution and lipid bilayer, respectively. All production simulations were conducted with the CUDA accelerated version of PMEMD module.

### 2.4 Partial nudged elastic band (PNEB) simulations

The nudged elastic band (NEB) method, developed as a chain-of-states approach, has been demonstrated to be an effective algorithm for investigating the pathways involved in the dynamic process of ligands access and binding (Bergonzo et al., 2011; Lu et al., 2014; Xing et al., 2019) as well as conformational changes of proteins in different states (Chen et al., 2014; Li et al., 2014). In NEB, a transition path is approximated with a string of images (or copies) of the system. Simultaneous energy minimization of the entire system by simulated annealing, but with the endpoint images fixed in space, generates a continuous minimum energy path. Each image in-between the two energy-minimized endpoints is connected to the previous and next image by springs along the path, which serve to keep each image from sliding down the energy landscape onto adjacent images. Prominently, a predefined reaction coordinate is not required to guide the generation of the minimum energy path in NEB simulations. Further details of the NEB method can be found elsewhere (Mathews and Case, 2006).

In this work, the partial nudged elastic band (PNEB) implementation (Bergonzo et al., 2009), a NEB variant specially suited for the large and explicitly solvated systems, combined with free energy calculations was used to explore the energetically favorable binding paths of the third-generation inhibitor tariquidar from the cytoplasmic gate region to the occluded central pocket and access tunnel site, respectively. The equilibrated structures of tariquidar-bound P-gp were assigned as the endpoint structures in the bound state. To obtain the endpoint structures of tariquidar in the pre-bound state, tariquidar was firstly docked into the apo structure of human P-gp (PDB ID: 7A65) using LeDock (Zhao and Caflisch, 2013; Shen et al., 2020). In the docking experiments, simulated annealing combined with the evolutionary algorithm was used to conduct an exhaustive conformational search for tariquidar in the cytoplasmic gate region, which was defined as a cubic box with a size of ca. 3.5 × 3.5 × 4.0 nm and a centroid of the surrounding residues including K181, S237, L245, E353, G360, A823, K877, E889, F994, and I1003. The obtained structures were embedded into a POPC lipid bilayer, solvated by TIP3P water molecules, and then neutralized with NaCl for the subsequent MD simulations, which were carried out with the aforementioned unbiased simulation parameters and MD protocols. The final snapshots were assigned as the endpoint structures in the pre-bound state.

PNEB calculations were conducted with the PMEMD.MPI module of AMBER18, according to the path optimization procedure shown in Supplementary Table S1. In all PNEB calculations, an NVT ensemble was used, and PNEB forces were applied on the TMDs to remove the rotational and translational motion. The initial binding path was constructed by 10 copies of the pre-bound endpoint structures and 10 copies of the bound endpoint structures. A total of 22 structures along the path were connected together by springs, and minimized simultaneously with the two endpoints fixed using a simulated annealing protocol (Supplementary Table S1). The initial path was heated from 0 K to 310 K over a period of 41 ps and equilibrated at 310 K for 200 ps using Langevin dynamics with a frequency of 1,000 ps^−1^ and a spring force constant of 10 kcal/mol/Å^2^. Then, a total of 1.8 ns of simulated annealing was performed to generate the consecutive minimum energy path, including 800 ps gradually heating the path to 510 K, followed by 600 ps slow cooling to 0 K, and finally 400 ps quenching dynamics to remove the remaining kinetic energy from the path using a spring force constant of 50 kcal/mol/Å^2^. 20 independent PNEB simulations were initiated from different random seeds for the initial Maxwell velocity distribution. A schematic overview of our simulation workflow is given in Supplementary Figure S1.

### 2.5 Binding free energy calculations using MM/GBSA method

Based on 20 snapshots of each image obtained from 20 independent PNEB simulations, the binding free energy profiles along the PNEB-optimized paths were calculated using the Molecular Mechanics Generalized Born Surface Area (MM/GBSA) method (Wang et al., 2019). Free energy differences are calculated by combining the gas phase energy contributions as well as solvation free energy components (both polar and non-polar) calculated using the Generalized Born solvent model. The free energy calculations were conducted with the Python script MMPBSA.py built-in AmberTools18 (Miller et al., 2012). POPC lipids, explicit water molecules and ions were stripped from the MD trajectories. Because our simulations show that no lipid molecules enter into the transport cavity and interact with the ligand, the lipid membrane was not included in MM/GBSA calculations. The dielectric constants of the protein and implicit solvent were set to 2 and 80, respectively. The salt concentration was set to 0.15 M. It must be here stressed that the numerical values of these calculated free energies were not expected to be exact, but rather to reveal relative likelihood of the tariquidar binding paths in human P-gp.

### 2.6 Analysis and visualization

All MD trajectories were post-processed and analyzed using the CPPTRAJ module built-in the AmberTools18 (Roe and Cheatham, 2013), unless stated otherwise. Atomic root-mean-square deviations (RMSD) were used to evaluate the equilibrium of the MD trajectories as well as the conformational changes of the simulation systems. The metrics such as distances and angles between specified atoms, residues, and domains were calculated to quantify the conformational dynamics and distributions of the protein and ligands in different states. In addition, the radius of gyration (R_gyr_), a descriptor of molecular size and shape, was used to characterize the molecular changes of tariquidar in different environments, which is calculated as the root-mean-square distance between the atoms of compound and its center of mass. All structural superposition were performed with the align function of PyMOL (the PyMOL Molecular Graphics System, Version 2.3 Schrödinger LLC.). Molecular graphics of the snapshots, trajectories and molecular interactions were prepared in PyMOL and VMD (Visual Molecular Dynamics, version 1.9.3).

## 3 Results

### 3.1 Revisiting the Cryo-EM structures of human P-gp: two binding sites for the third-generation inhibitors

**FIGURE 1:**
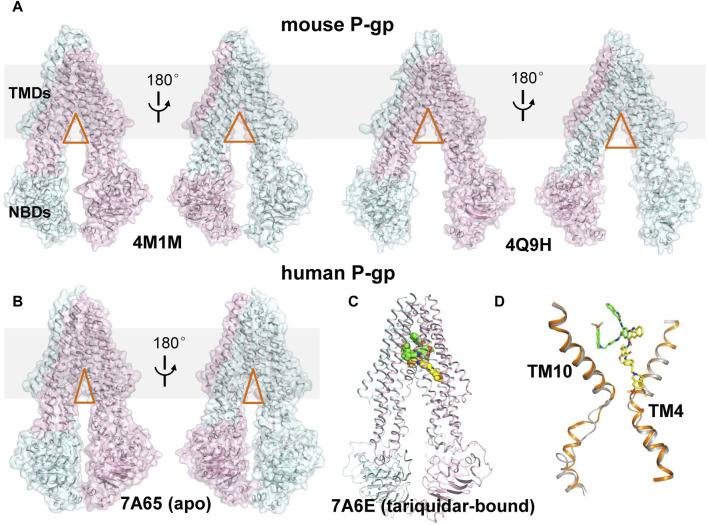
Structural comparison of mouse and human P-gp. **(A)** Cartoon and surface representation of the X-ray structures of mouse P-gp (PDB ID: 4M1M, 4Q9H). **(B)** Cartoon and surface representation of the cryo-EM structure of human P-gp in apo state. (PDB ID: 7A65, 7A6E). The N- and C-half of P-gp are shown in cyan and pink, respectively. The approximate position of a lipid bilayer is indicated by gray box. The opening extent of the proposed intramembranous gates are indicated by orange triangles. **(C)** Cartoon representation of the cryo-EM structure of human P-gp in tariquidar-bound state. The tariquidar molecules bound in the central pocket and access tunnel are shown as green and yellow spheres. **(D)** Structural comparison of TM4 and TM10 between the apo (gray) and tariquidar-bound (orange) structures of human P-gp. Tariquidar molecules bound in the transmembrane cavity are shown as green and yellow sticks.

Rapid advances in recent years have been witnessed in structural determination of P-gp in different conformational states, shedding light on the functional conformations during the transport cycle. Recently, the apo and cyclic inhibitor-bound structures of mouse P-gp are resolved by X-ray crystallography in a non-membrane environment (Aller et al., 2009; Ward et al., 2013), which adopt an inward-open conformation and reveal a spacious binding cavity within TMDs open to both the cytoplasm and the inner leaflet of the cell membrane, with a wide separation between the two NBDs (Figure 1A). These structures are frequently used to interpret the biochemical data and as templates to model the structure of human P-gp. However, whether these structures actually represent the physiological conformations in a membrane environment is still controversial. Importantly, designing inhibitors based on these structures has remained challenging, largely due to the featureless and poorly defined binding sites, together with the chameleonic behavior of the transporter in the absence of lipid membrane (Chufan et al., 2015).

More recently, cryo-EM structures of nanodisc-reconstituted, human P-gp in apo and drug-bound states have been determined, which are distinct from those of mouse P-gp (Alam et al., 2019; Nosol et al., 2020). These structures capture a homogeneous, inward-occluded conformation in which a drug-binding pocket at the center of the TMDs and a narrow cavity termed access tunnel site are formed by the kinking of transmembrane (TM) helices TM4 and TM10 toward the pseudosymmetric axis of the transporter, along with a concomitant closure of the two NBDs when compared with the inward-open structures of mouse P-gp (Figures 1B–D). The substrate-bound structures reveal a single anticancer drug (taxol or vincristine) bound in the central drug-binding pocket. By contrast, the third-generation inhibitors (tariquidar or elacridar) are bound in pairs, simultaneously occupying the drug-binding pocket and the access tunnel site (Figure 1C). One of the two inhibitor molecules, similar to the binding mode of taxol and vincristine molecules, adopts a folded U-shaped conformation fully enclosed in the drug-binding pocket. The second inhibitor molecule adopts an extended L-shaped conformation bound in the access tunnel site, which explains how the inhibitor prevents conformational transitions required for the drug efflux. Accordingly, it is assumed that a compound bound to the central binding pocket is likely to be a transport substrate, whereas the access tunnel is more prone to be a regulatory site.

However, tariquidar and elacridar have been reported to be dose- or concentration-dependently transported by human P-gp, thereby exhibiting dual characteristics as both substrates and inhibitors (Bankstahl et al., 2013). At high concentrations (micromolar level), they exhibit inhibitory effects by blocking P-gp-mediated drug transport, which can be explained by the cryo-EM structures. By contrast, at lower concentrations (nanomolar level), they can be recognized and transported by P-gp, therefore mimicking the behavior of substrates. This means that tariquidar selectively binds to the drug-binding pocket rather than the regulatory access tunnel at the single-molecule level, but the molecular mechanism remains unclear.

### 3.2 The occluded TMDs observed in human P-gp may represent an energetically favorable conformational substate

To unravel the selective binding mechanism of tariquidar at low concentrations, here, unbiased all-atom MD simulations of human P-gp were first performed to investigate the structural dynamics of human P-gp under a single inhibitor molecule bound state. To achieve this, four P-gp systems, i.e., two apo models and two tariquidar singly bound models, were independently simulated for 500 ns (duplicate in each case). For simplicity, these simulation systems are hereafter referred to as APO1 (from the cryo-EM structure of P-gp in drug-free state), APO2 (from the cryo-EM structure of tariquidar-bound P-gp), TCP (tariquidar singly bound in the central pocket), and TAT (tariquidar singly bound in the access tunnel), respectively (Table 1). The simulation equilibrium and conformational changes of P-gp were preliminarily evaluated by the RMSD of backbone Cα atoms with respect to the starting structures (Supplementary Figures S2, S3). Overall, the small fluctuation of Cα-RMSD in the last 300 ns simulations (4.28 ± 0.22 Å and 3.60 ± 0.21 Å for APO1, 3.91 ± 0.15 Å and 5.05 ± 0.26 Å for APO2, 3.55 ± 0.19 Å and 6.20 ± 0.2 Å for TCP, 8.48 ± 0.49 Å and 4.25 ± 0.28 Å for TAT) demonstrates that all systems reached equilibrium, and at equilibrium states, both of the apo and tariquidar-bound P-gp underwent conformational changes in one of the simulated replicas. The major conformational changes are mainly attributed to the high dynamic in the NBD dimer, with a dramatical change in the RMSD value of NBDs (∼4–13 Å). In comparison, no significant changes in the RMSD value of TMDs (∼2–4 Å) are observed across all of these simulations, whether in the presence of tariquidar or not, indicating that the conformation of TMDs remains relatively stable.

**TABLE 1:** Details of the systems and unbiased simulations performed in this study.

System	Ligand states	No. of replicas	Time (ns) per replica
Human P-gp systems			
APO1^a^	No ligands	2	500
APO2^b^	No ligands	2	500
TCP	tariquidar bound in the central pocket	2	500
TAT	tariquidar bound in the access tunnel	2	500
Single tariquidar systems			
L-shaped conformer	Free in water	3	500
L-shaped conformer	Free in membrane	3	500
U-shaped conformer	Free in water	3	500
U-shaped conformer	Free in membrane	3	500

^a^ APO1 was modeled based on the cryo-EM structure of human P-gp (PDB ID: 7A65) in drug-free state.

^b^ APO2 was modeled based on the cryo-EM structure of tariquidar-bound human P-gp (PDB ID: 7A6E) after removing all ligands.

**FIGURE 2:**
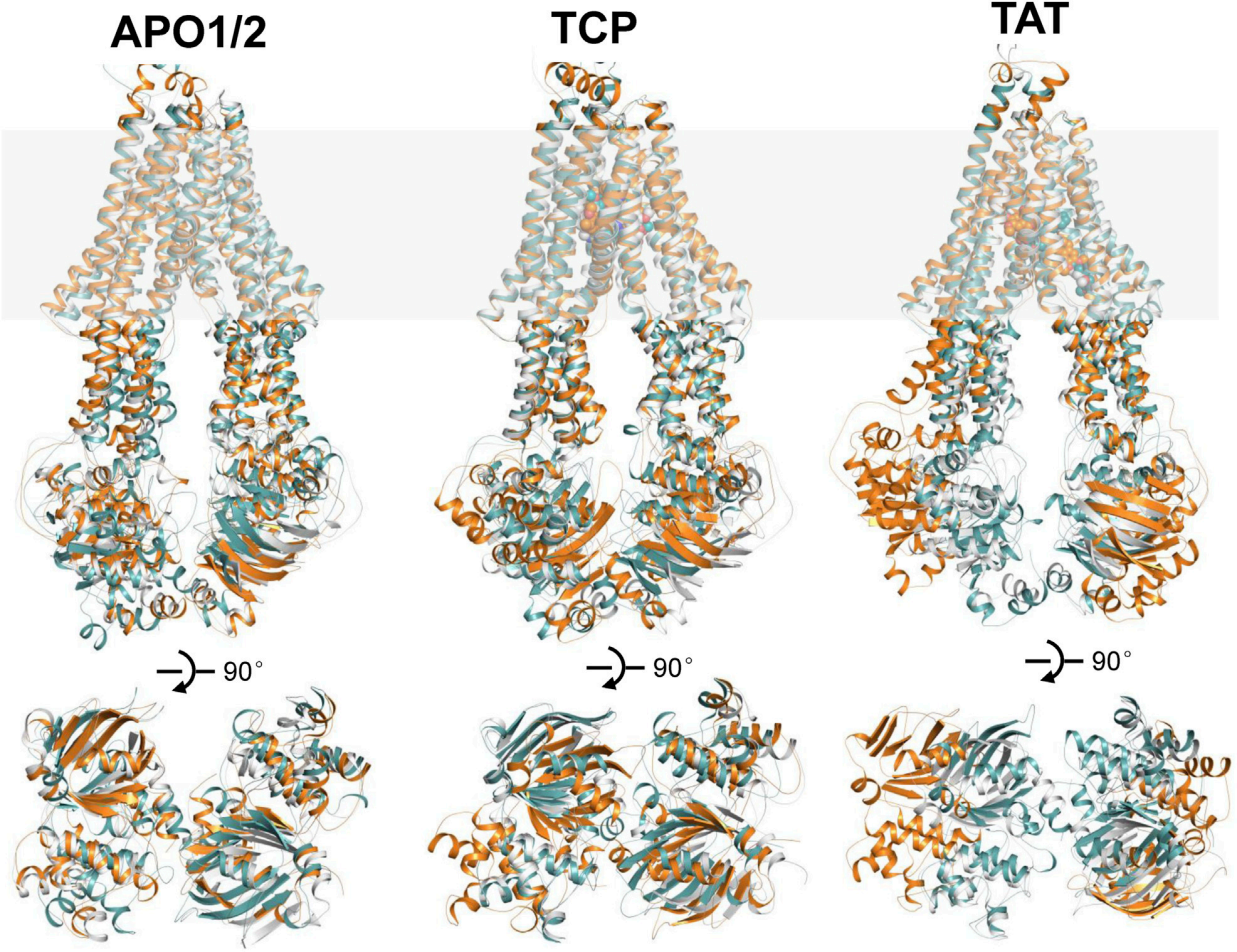
Structural alignments of the snapshots after 500 ns MD simulations of APO1/2, TCP and TAT onto the cryo-EM structures of human P-gp in apo and singly tariquidar-bound states. (Top panel) Cartoon representation of the MD snapshots (cyan and orange) and cryo-EM structures (white). The approximate position of a lipid bilayer is indicated by gray box. Tariquidar molecules bound in the transmembrane cavity are shown as spheres. (Bottom panel) Extracellular view of the NBDs after structural superposition of TMDs from the extracellular side. For clarity, TM helices are not shown.

**FIGURE 3:**
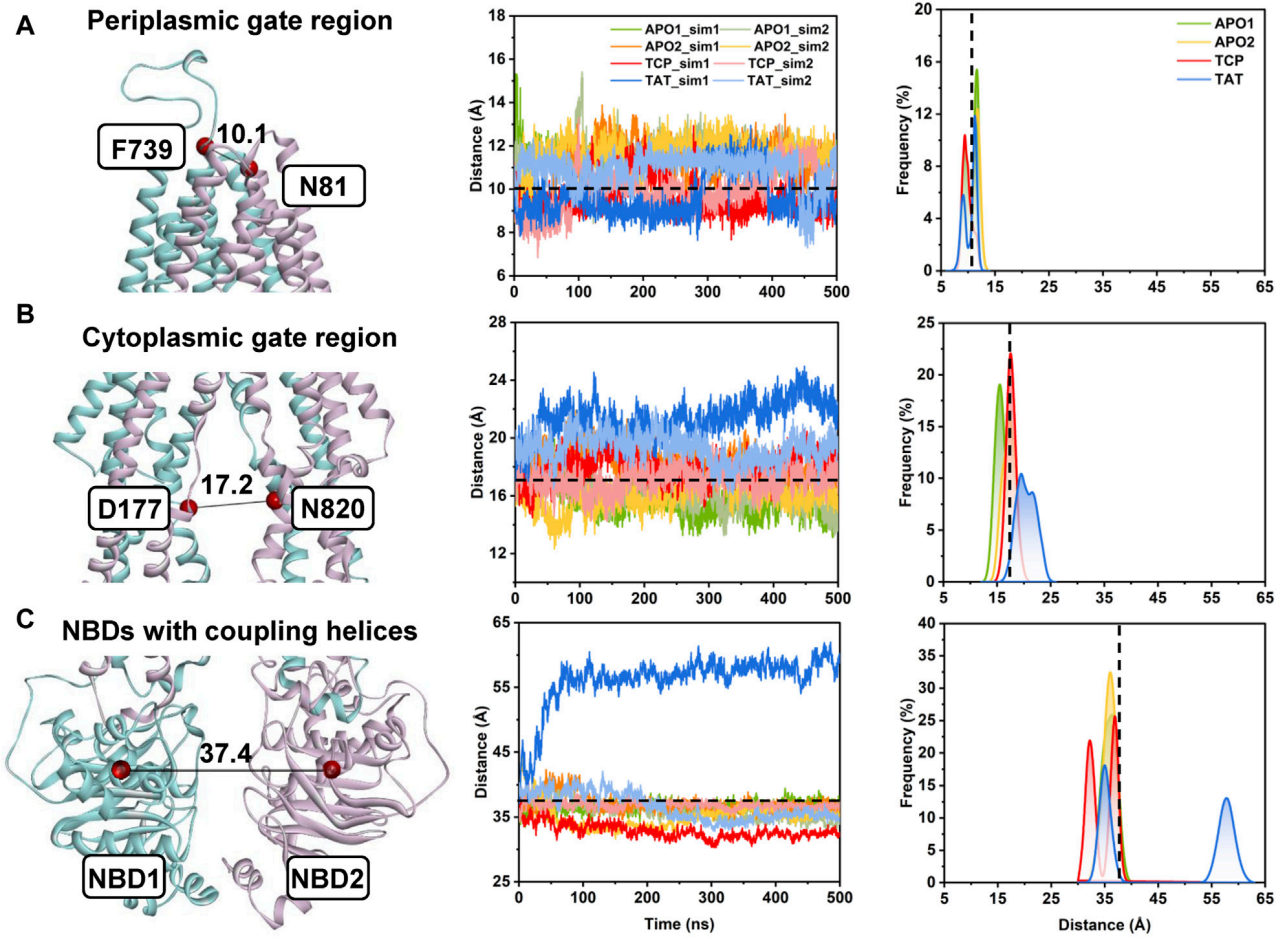
Conformational dynamics of human P-gp from unbiased MD simulations. **(A)** The distance between the Cα atoms of residue pair N81TM1−F739TM7 indicates the conformational dynamics of the periplasmic TMD gate region. **(B)** The distance between the Cα atoms of residue pair D177TM3−N820TM9 indicates the conformational dynamics of the cytoplasmic TMD gate region. **(C)** The distance between the center of mass of the NBD1 (residues L392 to K619) and NBD2 (residues V1035 to H1254) indicates the opening/closing of the NBD dimer. (Left panel) Cartoon representation of apo human P-gp (PDB id: 7A65) with the initial distances highlighted in black lines. (Middle panel) Time series of the distances from the MD simulations. (Right panel) Distance distributions from the equilibrium states of both simulated replicas (i.e., from 200 to 500 ns) for each system. Initial distances in the cryo-EM structure are indicated by black dashed lines.

In the following, the global conformational dynamics of P-gp were described in more detail. Figure 2 shows snapshots obtained from our MD simulations and structural superposition with the starting structures. Compared to the APO and TCP systems, simulations of TAT system captured two distinct conformations with tariquidar bound at the access tunnel, one featuring a wider gap between the NBDs, and the second featuring semi-closed NBDs similar to that in the cryo-EM structures of the transporter. To characterize the global conformational changes in more detail, the distances between the center of mass of the NBD pair, along with the Cα atoms of residue pairs in the cytoplasmic (intracellular) and periplasmic (extracellular) TMD gate regions were monitored, respectively (Figure 3). In all simulations, the Cα−Cα distance between the residues pair in the extracellular side fluctuates around 10 Å, indicating that the periplasmic TM gate stays closed in all simulation systems as exhibited in the cryo-EM structures (Figure 3A). The previously proposed entrance gates still remain semi-closed (or occluded) in the apo state, as indicated by the decreased distances between the residue pair in the intracellular side of the TMDs (Figure 3B). By contrast, a slight opening movement of the cytoplasmic TM gate region can be observed in one of the two TAT simulations. The appearance of a prominent increase in the NBD distance reveals concomitant opening of the NBD dimer when tariquidar singly bound at the access tunnel (Figure 3C), consistent with the structural observation from our simulations (Figure 2). It is also important to note that the NBD distance in most of the simulations (7 out of 8) shows a decrease in various degrees, especially when tariquidar bound in the central drug-binding pocket (Figure 3C), indicative of a moderate closure of the NBDs relative to the cryo-EM structures.

### 3.3 Both transmembrane sites remain stable while exhibiting a dynamic binding mode at the single-tariquidar level

**FIGURE 4:**
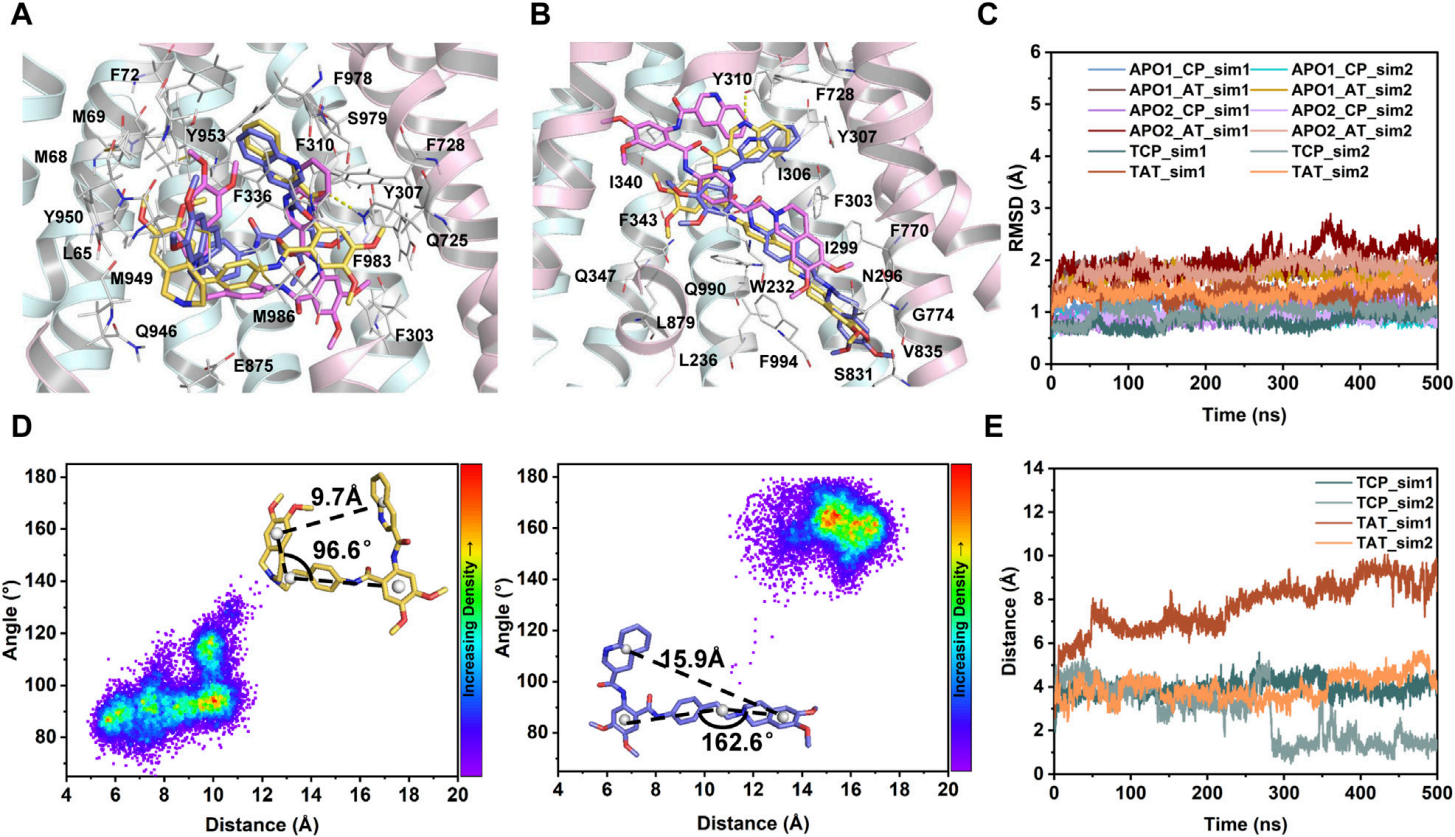
MD characterization of dynamics in the transmembrane sites. **(A)** Close-up view of the central binding pocket. **(B)** Close-up view of the access tunnel site. For clarity, only the cryo-EM structure of human P-gp (PDB id: 7A6E) is shown as cartoon. Tariquidar molecules from the cryo-EM structure (yellow) and the last MD snapshots (magenta and purple) from two simulated replicas are shown as sticks. Residues within 3 Å of tariquidar are shown as white sticks and labeled. Hydrogen bonds observed in the cryo-EM conformation are shown as yellow dashed lines. **(C)** Time evolution of Cα-RMSD of the residues lining the central pocket (CP) and access tunnel (AT) during the courses of 2 × 500 ns MD simulations for each system. **(D)** Conformational stability of tariquidar *per se* in the binding sites indicated by two-dimension distribution of the distance and internal angle indicated in the insets. **(E)** Time series of the distance between the center of mass of tariquidar and the contacting residues in the corresponding binding site.

To further characterize the dynamics in the binding sites and the binding stability in the presence of a single copy of tariquidar, the conformational dynamics of both sites was quantified by RMSD calculations during the course of duplicate 500 ns simulations of each system (Figures 4A–C). The Cα-RMSD of the residues contacting with tariquidar in each site remains, on average, at ∼1.5 Å across all simulations, indicating that both sites are stable, with a negligible difference in the apo and singly tariquidar-bound states (Figure 4C). Significant conformational transitions in tariquidar *per se* are not observed in both sites, indicating that the folded, U-shaped and extended, L-shaped conformations adopted by tariquidar are stable in our MD simulations (Figure 4D). However, tariquidar bound in the access tunnel site shifts upward to the central pocket, and alterative molecular orientation in the central pocket is also observed from one of the two simulated replica for TAT and TCP system (Figures 4A, B, E), indicative of moderate binding of tariquidar in both transmembrane sites at the level of the individual molecules. These results are consistent with the cryo-EM observations in which the EM densities for the bound molecules are not well defined, even though the density allowed confident fitting of the molecular scaffold.

Together, it can be inferred that the occluded TMDs, as presented in all the substrate- and inhibitor-bound human P-gp, may be trapped in a deep free energy minimum and represent a druggable conformation in the transport cycle. However, the dynamic binding mode and alterative orientation of the single tariquidar bound at both transmembrane sites may be in favor of the subsequent transport, which reduce the potential to prevent the conformational changes. The binding and hydrolysis of ATP molecules is required to drive further conformational transitions from the occluded to the outward-open states (Kim and Chen, 2018).

### 3.4 Lipid membrane may play a role in preconfiguring the favorable conformation of tariquidar for access and binding to the transmembrane sites

Knowledge on the conformational preferences and dynamics of the free ligand could guide rational drug design. Tariquidar is a highly lipophilic molecule with a calculated logP value of 6.38 and has a large number of rotatable bonds (nRotB = 11), which leads to a rather broad range of accessible conformers. However, little is known about the conformational preference of tariquidar in membrane and its impact on the binding to the transmembrane sites. Hence, to further investigate the entry mechanism, we examined the dynamics, conformational space, and the preferred conformation of tariquidar in aqueous solution and lipid membrane by unbiased MD simulations. To enhance sampling, both L- and U-shaped conformations of tariquidar extracted from the cryo-EM structures were used to initiate six independent simulations, each of length 500 ns, yielding a total of 120,000 snapshots of tariquidar for water and membrane phases, respectively.

**FIGURE 5:**
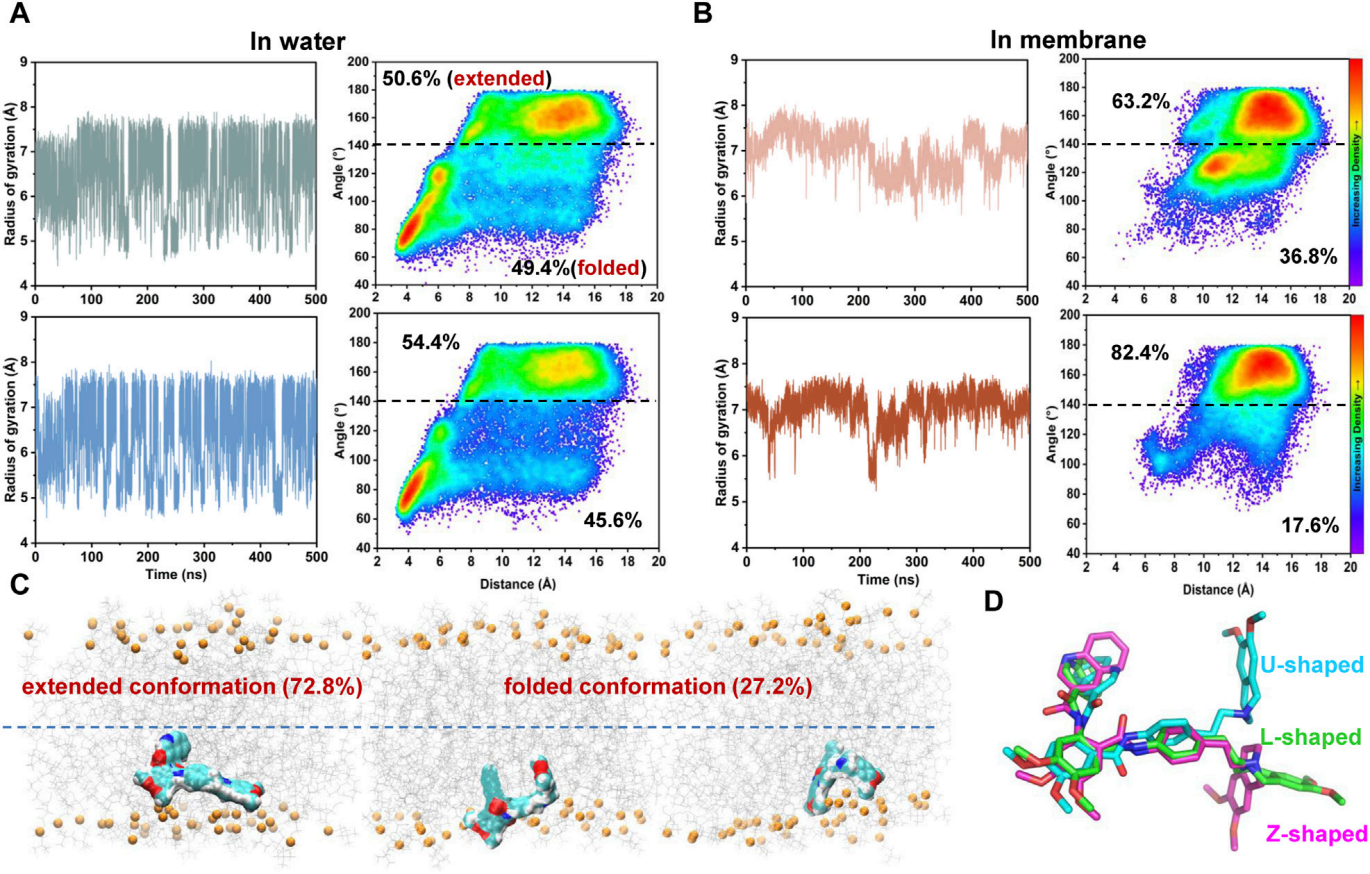
Environment-dependent conformational preference of tariquidar. **(A)** Simulation of tariquidar in water. (Left) Time evolution of the radius of gyration (Rgyr) of tariquidar. (Right) Conformational ensembles of tariquidar generated by a total of 3 μs MD simulations in water. **(B)** Simulation of tariquidar in lipid membrane. (Left) Time evolution of the radius of gyration (Rgyr) of tariquidar. (Right) Conformational ensembles of tariquidar generated by a total of 3 μs MD simulations in lipid membrane. In a and b, three simulations of tariquidar initiated with the folded conformation is shown in the upper panel, and the other three with the extended conformation is shown in the lower panel. **(C)** The tariquidar conformation and orientation in lipid membrane. The population of each state is indicated. **(D)** Representative conformations of tariquidar showing the flexibility of the tetrahydroisoquinoline ring.

Simulations of tariquidar in water reveal quick conformational transitions between the folded and extended states, as manifested by the RMSD (Supplementary Figure S4) and radius of gyration (R_gyr_) of tariquidar (Figure 5A; Supplementary Figure S5). To access the most preferred conformation, the conformational space of tariquidar observed from the 1.5 μs MD simulations was characterized by the 2D distribution of the distance and internal angle shown in Figure 4D. By inspecting all the MD-generated snapshots, we found that the internal angle greater than 140° indicates an extended conformation, and the remaining can be categorized into the folded conformational subset, although with a broad distance distribution between the end aromatic groups of tariquidar. The extended and folded conformations are sampled equivalently in water (Figure 5A). In contrast, tariquidar adopts a relatively extended conformation in most of the snapshots (∼73%) sampled in membrane, which is almost identical to the L-shaped conformation bound in the access tunnel site of human P-gp (Figures 4B, 5B). In addition to the U-shaped conformation bound in the central pocket, we also observed another folded conformation, hereafter referred to as Z-shaped, in the conformational spaces in both phases, as a result of the rotation of the tetrahydroisoquinoline ring around the hydrophobic ethyl linker (Figure 5D). By comparison, the extended, L-shaped conformer is flatter with reduced dimensionality, which passes the previously proposed intramembranous gates more easily. Furthermore, the compound is mostly coordinated near the bilayer headgroups via polar interactions such as H-bonds between the amide group of tariquidar and phosphate groups of the lipids (Figure 5C). Thus, the preferred conformation and location in lipid membrane supports the gating mechanism by which tariquidar penetrates into the transmembrane cavity of P-gp through intramembranous gates. These findings suggest that the lipid membrane not only acts as a reservoir to increase the local concentrations of the lipophilic tariquidar near the gate regions, but also plays a critical role in preconfiguring its orientation and conformation favorable for traversing the lateral gates.

### 3.5 PNEB simulations reveal two conformation-dependent pathways for tariquidar entry into to the central drug-binding pocket

**FIGURE 6:**
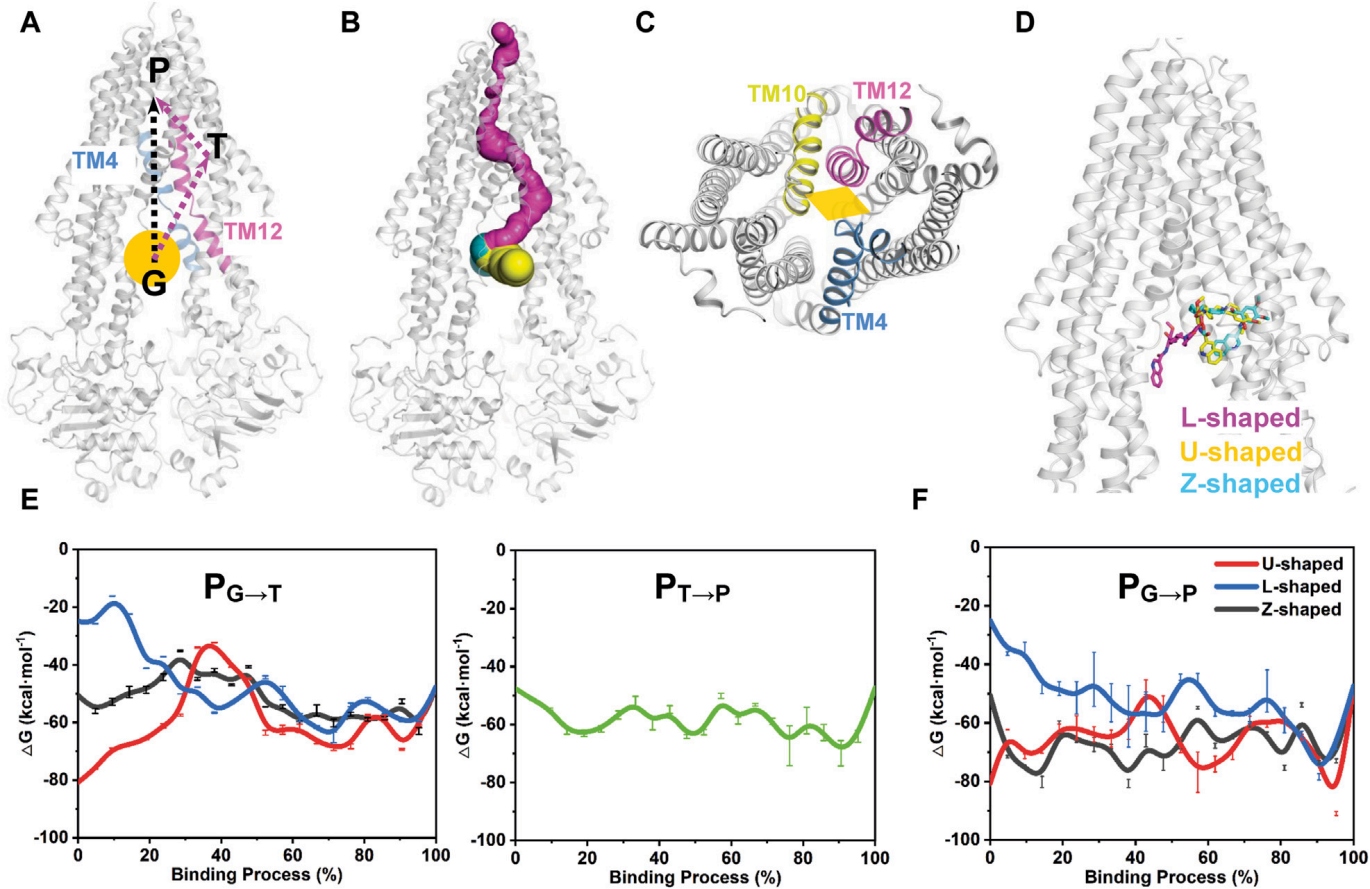
Free energy profiles of tariquidar binding along the putative pathways with different starting points. **(A)** The two potential binding paths proposed in this work are indicated by black and purple arrows. The gate region is pointed by an orange ball. G, gate region; T, access tunnel; P, central pocket. **(B)** Path detection by CAVER 3.0 software with a 0.9 Å probe radius, 3 Å shell radius, and 4 Å shell depth. The detected path from the gate region to the central pocket via the access tunnel is shown in purple. The two proposed drug-entry portals laterally open to the membrane are shown in yellow and cyan, respectively. **(C)** The cytoplasmic entrance gate formed by the kinking of TM4, TM10 and TM12 is indicated by an orange diamond. **(D)** The starting point structures with different conformations adopted by tariquidar shown as sticks. **(E, F)** The free energy profiles for tariquidar entering into the transmembrane sites along the PNEB-generated paths.

Cryo-EM structures have revealed a dually tariquidar-bound mode of inhibition, but the molecular mechanism by which tariquidar selectively bind to the central pocket and is transported at low concentrations remains poorly understood. Therefore, we carried out atom-level simulations to elucidate the pathways involved in the dynamic process of the lipophilic tariquidar reaches the transmembrane sites of P-gp, originating from the cytoplasmic gate region situated at the bottom of the central cavity (Figure 6A). Both kinetic and thermodynamic information about the ligand binding processes can be assessed by calculating the free energy profile or potential of mean force along a predefined reaction coordinate that describes the process. However, the time scales accessible in unbiased MD simulations are typically too short to characterize such slow processes, and the choice of an adequate reaction coordinate is challenging for complex membrane protein system, especially for ABC transporters. To circumvent these limitations, we employed the time-independent partial nudged elastic band (PNEB) method to generate the minimum energy pathway (MEP) depicting the binding of tariquidar to the central pocket and access tunnel site. Notably, the PNEB method obviates the need for a pre-assumed reaction coordinate to guide MEP generation, ensuring that the path between two endpoints remains continuous in all solute degrees of freedom.

In this work, two putative access and binding pathways are explored (Figure 6A). An upward movement of tariquidar bound in the access tunnel was observed from our unbiased MD simulations, suggesting a potential binding path (P_G→T→P_) that starts from the gate region, then traverses the access tunnel, and finally reaches the central pocket (Figure 6B), which is consistent with that detected by CAVER 3.0 software (Chovancova et al., 2012). An alternative path (P_G→P_) by which tariquidar enters into the central pocket directly via the gate formed by the kinked TM4, TM10 and TM12 is also proposed and checked (Figures 6A, C). Thus, both paths were generated by PNEB simulations and evaluated by binding free energy (ΔG) along the corresponding path that were calculated by MM/GBSA method. Prominently, to examine the conformational effects of tariquidar on the binding process, the starting structures of tariquidar docked in the cytoplasmic gate region were modeled by molecular docking (Supplementary Table S2; Supplementary Figure S6), and then equilibrated by 500 ns MD simulations. The transporter and the binding of tariquidar in the pre-bound state reach equilibrium quickly, and remain stable within the remaining 300 ns simulations (Supplementary Figure S7). Therefore, the last snapshots of the three independent simulations were used as the starting point structures for PNEB calculation where tariquidar adopted L-, U- and Z-shaped conformations, respectively (Figure 6D).

In both PNEB-optimized paths, the access of the folded tariquidar (U- and Z-shaped) shows a striking increase in the binding free energy and encounters a relatively high energetic barrier before reaching the central pocket and the access tunnel, respectively (Figures 6E, F). By contrast, the extended L-shaped tariquidar passes through the gate region and then enters into the corresponding site with progressively decreased binding free energies. More interestingly, the free energy landscape of tariquidar binding to the central pocket from the access tunnel remains relatively flat, indicating a spontaneous penetration process (Figure 6E). Therefore, these results demonstrate the existence of the two conformation-dependent binding paths, at least with respect to tariquidar, where the extended conformer predominantly adopted in the lipid membrane are more favorable for the entry into the transmembrane sites.

**FIGURE 7:**
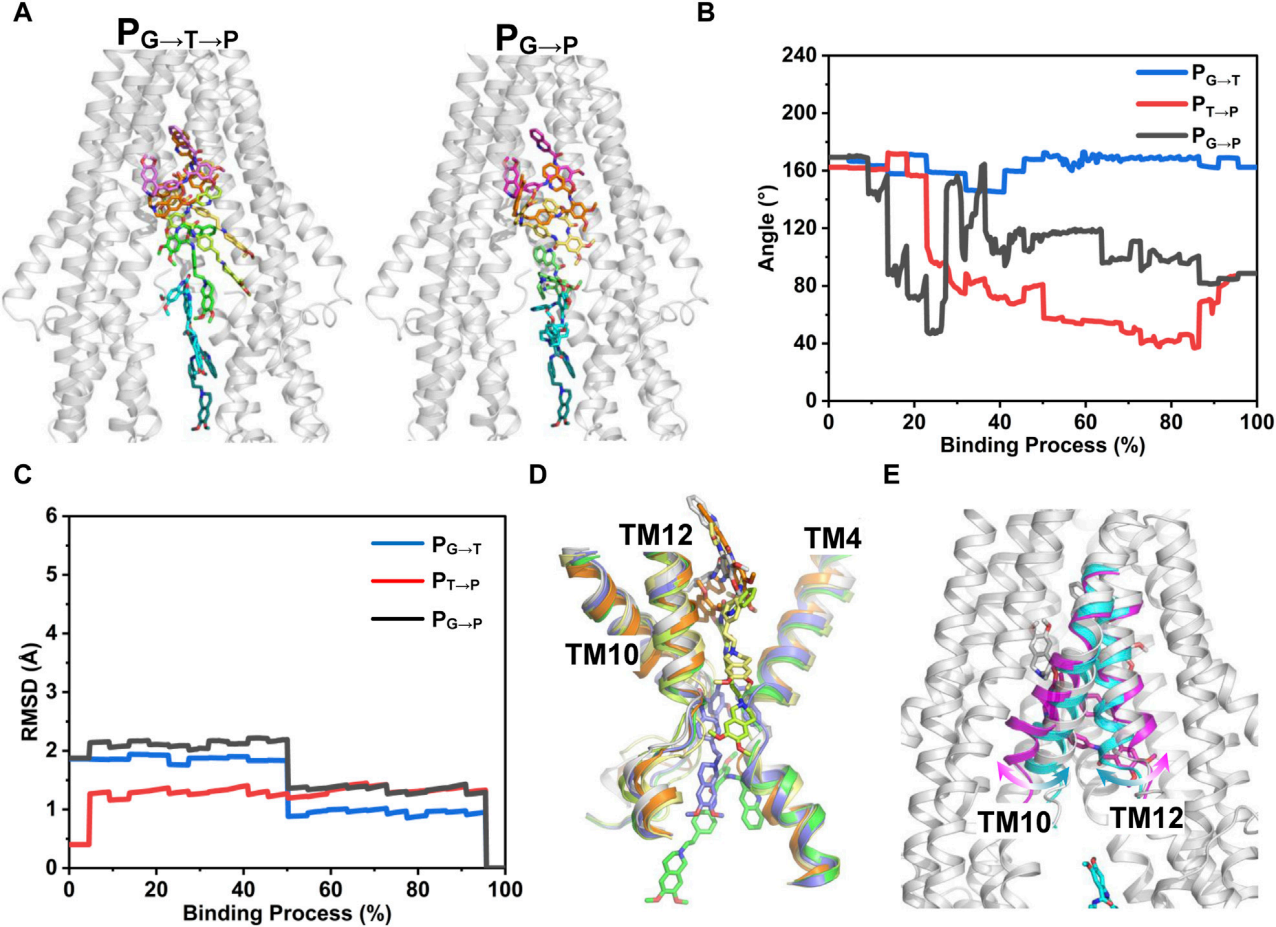
PNEB-optimized pathways. **(A)** Front view of the two time-independent binding paths of tariquidar are indicated by snapshots. Tariquidar is shown in sticks. For clarity, TM 10 and TM12 are not shown. **(B)** Conformational transitions of tariquidar along the binding paths indicated by the internal angle as shown in Figure 4. **(C)** Conformational changes of P-gp TMDs along the binding paths indicated by the Cα-RMSD. **(D, E)** Conformational changes of the gate-involved TMs in P_G→T→P_ **(D)** and P_G→P_ **(E)**.

Figure 7 shows the time-independent tariquidar-binding paths and the associated conformational changes of the protein and ligand along the corresponding path. To describe the pathways in more detail, the pivotal tariquidar-interacting residues in both pathways are labeled and shown in Supplementary Figure S8. The two PNEB-optimized paths are similar in appearance, both of which traverse narrow cleft between the kinked TM4 and TM12 and finally reach the central pocket (Figure 7A). However, distinct conformational changes of tariquidar are observed in the two binding processes. In P_G→T→P_, the extended conformation is maintained until reaching the narrow tunnel site, and then transformed into the folded conformation to accommodate the globular central pocket (Figure 7B). By contrast, in P_G→P_, a quick conformational transition between the extended and the folded states is observed when tariquidar passed through the gate region, largely due to the flexible rotation of the tetrahydroisoquinoline ring. In both paths, significant conformational changes in the TMDs of the transporter are not observed (Figures 7C–E). However, the TM10 and TM12 undergo local movements in P_G→P_, allowing tariquidar entering into the central pocket with a folded conformation (Figure 7E). These findings indicate that the conformational flexibility of tariquidar is highly favorable to the ultimate binding to the central pocket in both pathways.

## 4 Discussion

P-gp is one of the most promiscuous multidrug transporters which plays a vital role in determining the ADMET profile of therapeutics, and contributes to MDR in cancer, thus becoming a biomedically important target. With the significant progress in crystallization and structural determination of membrane proteins, several cryo-EM structures of human P-gp complexed with a single chemotherapeutic agent or a pair of third-generation inhibitors have been reported recently. These structures provide novel insights into the drug binding and inhibition mechanisms of P-gp. Dissimilar to the previously reported mouse P-gp with a large and featureless binding cavity, the structures of human P-gp are trapped in an inward-occluded state in which two transmembrane sites, i.e., the central pocket and access tunnel, formed by the bending and kinking of TM4 and TM10, are well profiled (Nosol et al., 2020). Typically, the high-affinity third-generation inhibitor, tariquidar, prevents the conformational transitions required for the drug efflux through a dually bound manner at higher concentrations. Interestingly, it acts as a transport substrate at low concentrations. A detailed understanding of the molecular mechanism underlying the tariquidar-induced activation and inhibition of P-gp transport is crucial for rational design of novel therapeutics. To this end, the structural dynamics of human P-gp as well as the access and binding mechanisms at the level of the individual tariquidar molecule were investigated by all-atom MD simulations in this work. Our key findings include: (i) The transmembrane sites within the occluded TMDs of human P-gp remain stable in the presence or absence of a single copy of tariquidar. (ii) The membrane lipids pre-organize tariquidar in location and conformation favorable for access and binding to the transmembrane sites. (iii) Multiple binding pathways, at least two for tariquidar, exist in the translocation cavity. These findings not only provide molecular explanation for how tariquidar selectively binds to the drug-binding pocket and is transported at low concentration but also help the development of more effective P-gp inhibitors.

Previous researches have suggested the existence of multiple binding sites in the transporter cavity, such as the H-site (Hoechst-33342 binding site) and R-site (Rhodamine-123 binding site) accounting for polyspecificity (Martinez et al., 2014). However, a structural definition of the proposed binding sites is lack, limiting rational drug design. Thus, capturing a stable P-gp structure with tractable binding sites is pivotal for targeted inhibition of P-gp. The recent cryo-EM structures of human P-gp, for the first time, offer unprecedented molecular details on the existence of the drug-binding pocket and a specific access tunnel site where small molecules likely bind and modulate the transporter. However, these structures were determined with the help of the antigen-binding fragment (Fab) of the inhibitory antibody UIC2 or MRK16 at higher compound concentrations. Additionally, the inherent flexibility of the transporter limits local resolution and the elucidation of molecular interactions in the cryo-EM models. To gather atomistic insight into inhibitor binding at single-molecule level, as well as the resulting dynamics in the protein structure and binding sites, we carried out MD simulations of human P-gp without any monoclonal antibodies.

A set of unguided simulations showed that the occluded TMDs remains stable in all modeled states, although relatively large fluctuations are observed in the kinked TM4 and TM10 involving in the formation of the putative and cytoplasmic portal for drug entry into the transmembrane sites (Supplementary Figure S9). Site-directed mutagenesis experiments have demonstrated that the mobility or flexibility of the TM4 and TM10 is critical for efficient drug binding and transport (Kodan et al., 2014; Rahman et al., 2023). Both transmembrane sites and the bound tariquidar *per se* show stable conformations in all simulated replicas (Figure 4). However, in the singly tariquidar-bound states, the folded and extended conformers bound in the corresponding site showed orientational and positional flexibility (Figure 4). In the cryo-EM structure, tariquidar binds in pairs, where the two conformers interact with each other by the adjacent aromatic rings and amide-amide interactions that can strengthen their binding stability (Figure 1D). These intermolecular interactions would be disrupted once either of them is removed, resulting in the impairment of binding stability. As observed in the vincristine-bound structure, the EM density for vincristine bound in the central pocket is also poorly defined, indicating alternative orientations. It is therefore further speculated that a loose binding of vincristine or tariquidar in the central pocket may be beneficial to the following transport driven by ATP hydrolysis. Intriguingly, a distinct conformational basin with a relatively large separation between the NBDs was sampled when a single tariquidar bound in the access tunnel, suggesting a distal allosteric regulation to hinder the dimerization of the NBDs required for ATP hydrolysis, as discussed in the previous study (Dastvan et al., 2019). Pharmacological researches have shown a non-competitive interaction between tariquidar (XR9576) and the P-gp substrates vinblastine and paclitaxel, suggesting that at least two distinct sites serving regulatory and transport functions coexist in P-gp (Martin et al., 1999; Martin et al., 2000). This supports the notion that the access tunnel is likely to be a regulatory site that could be occupied by small-molecule compounds in an extended conformation while the central pocket binds anti-cancer drugs for transport. However, a slight movement of the extended tariquidar toward the central pocket can be observed from our simulations, hinting at a potential transport when bound at the access tunnel. Thus, we concluded that the ligand size and 3D shape may be important molecular determinants for the binding stoichiometry and stability of small-molecule inhibitors targeting P-gp. A single compound with a large size may be better suited to match the shape of the two binding sites, and form H-bonds interactions dominant in transport inhibition, further allowing a more confined binding and thus reducing the possibility of transport.

The apo and ligand-bound holo structures of human P-gp are captured in an inward-occluded state and no significant structural differences are observed among them, leaving an important question as to how tariquidar molecules access and bind to the occluded central pocket and the regulatory site. It is well established that ligand binding from water to the transmembrane cavity of P-gp includes a lipid-water partitioning step followed by a cavity-binding step in the lipid membrane (Xu et al., 2015). As such, the lipid membrane likely plays an important role in modulating the drug interactions with the P-gp, but the molecular details remain largely unknown. A recent computational investigation on the mouse P-gp proposed a lipid-synergetic inhibitory mechanism for tariquidar in which lipid molecules penetrated into the large cavity to help the restriction of the inward-open conformation when a single tariquidar bound in the central pocket (Kapoor et al., 2021). As discussed by the authors, the two proposed drug-entry portals existing in the mouse P-gp TMDs are enough wide to allow the lateral translocation and diffusion of the lipid molecules into the central cavity. However, the penetration of lipids has yet been observed from either our MD simulations in singly tariquidar-bound state or the cryo-EM structures of human P-gp bound two tariquidar molecules determined in the near physiological conditions, i.e., the nanodiscs, which suggests that the membrane lipids do not participate directly in the inhibition of the transporter. Additional roles may be served by the lipid membrane in the access and binding process of tariquidar to human P-gp. Considering the conformational heterogeneity of tariquidar, we investigated the role of lipid membrane in modulating the ligand conformation and its impact on the binding to the transmembrane sites. Extensive μs-long all-atom simulations of tariquidar in different environments revealed a dynamic equilibrium between the folded and extended states in water solution, and a significant shift of the equilibrium towards predominantly extended conformations in the lipid membrane (Figure 5). The preferred orientation of the ligand is close to the bilayer headgroups, which increases the chance of access to the entry portals that are open to the low leaflet of the membrane. Furthermore, the extended conformer is flatter, and thus shows less dimensionality than the folded one and the lipid molecules, which is expected to make the diffusion of tariquidar into the cavity easier through the relatively narrow portals existing in the occluded TMDs of human P-gp. This point is supported by a computational study which revealed a spontaneous translocation process of tariquidar through the putative entrance gate in an extended conformation (Ferreira et al., 2015). More interestingly, the preferred conformation of tariquidar within the lipid bilayer is strikingly similar to that of the ligand bound in the access tunnel site bearing a modulatory role. The previous quantitative structure-activity relationship study has suggested that the extended conformation of tariquidar and its derivatives is closely correlated with their inhibitory activity (Labrie et al., 2006). In earlier studies, membrane-mediated generation of bio-active conformation of amphiphilic peptides has been proposed as an important role of the lipid membrane in catalyzing peptide-receptor interactions (Sargent and Schwyzer, 1986; Schwyzer, 1995). Taken together, we propose that the lipid membrane could pre-organize tariquidar in an active conformation that is preferable for the binding to the regulatory site, and thus exerting inhibitory function.

To further substantiate our speculation, we explored the dynamic process involved in tariquidar entry into the transmembrane sites from the cytoplasmic gate region at atomistic level. PNEB simulations revealed dual asymmetric binding pathways, i.e., P_G→T→P_ and P_G→P_ for tariquidar within the TMDs (Figure 7). In both paths, the extended conformation initially adopted by tariquidar in the lipid membrane is more energetically favorable (Figure 6), reinforcing the notion that the lipid membrane has a critical role in preconfiguring tariquidar molecules in an optimal orientation and conformation for the access and binding to the transmembrane sites. Recent biochemical and computational studies have demonstrated the coexistence of multiple drug translocation pathways in P-gp cavity, one of which is preferentially, but non-exclusively, taken by a single substrate or inhibitor molecule (Parveen et al., 2011; McCormick et al., 2015; Subramanian et al., 2019). The two binding pathways identified from our simulations are distinct but overlapping partially, which can be distinguished by the orientations (or conformations) of tariquidar in the translocation cavity. In the case of P_G→T→P_, the ligand firstly penetrates into the regulatory site (access tunnel) adopting an extended conformation throughout the access process, and then enters into the central pocket spontaneously with a conformational transition to the folded conformation. By contrast, the other pathway, P_G→P_, is close to the pseudosymmetric axis of the transporter in which tariquidar enters directly into the central pocket from the cytoplasmic gate region bypassing the access tunnel site, with a quick conformational transition between the extended and the folded states as well as a concomitant motion in TM10 and TM12, which agrees with, and to some extent, explains the proposed induced-fit mechanism for drug binding (Loo et al., 2003). The conformational transitions of tariquidar occurred in both translocation pathways, which is also observed from the cryo-EM structures of another multidrug ABC transporter, ABCG2, in different tariquidar-bound states, indicates that the ligand flexibility is a major contributor to the selective binding to the central drug-binding pocket (Rasouli et al., 2023). Furthermore, the previous study has shown that mutation of the residues Y307 and Q725, which are located in the P_G→T→P_ identified from our simulations, led to the loss of the inhibitory activity of tariquidar for the photolabeling of a P-gp substrate [125I]-iodoarylazidoprazosin (IAAP); however, the ATP hydrolysis of the mutant P-gp can be still modulated by tariquidar (Chufan et al., 2013), suggesting that P_G→T→P_ is likely to be the primary binding pathway while the P_G→P_ is the second one. The dual binding pathways revealed in this work can explain how tariquidar selectively bind to the central pocket for transport at the level of an individual molecule. Of note, we cannot exclude other binding pathways or mechanisms when the transporter is trapped in another metastable and distinct conformations in the transport cycle.

It should be also noted that there are limitations in this study. First, the simulations of each P-gp system were performed only with two replicas, potentially resulting in limited sampling. Although a different conformational state with a large separation between the NBDs was sampled, more simulation replicas may be required to further verify this observation. Second, the membrane model used here is a single POPC bilayer. Although a pure membrane model is usually used in ABC system simulations, it is relatively simple and different from the real environments. Membrane proteins such as P-gp are involved in complex relationships with the lipid environment. Therefore, the impacts of the lipid type and membrane compositions on the P-gp dynamics and ligand binding could be further evaluated. However, the specific effect of membrane compositions (mixtures of saturated and unsaturated lipids as well as cholesterol) on the structure and functions of P-gp remains a challenging question so far. Additional studies are needed to elucidate this point. Third, we used PNEB and MM/GBSA calculations to explore the energetically favorable ligand entry pathways in the TMDs. This approach has been proved to be an effective algorithm to characterize the pathways involved in the dynamic process of ligands access and binding (Lu et al., 2014; Xing et al., 2019). The free energy along the pathways was roughly estimated without a consideration of entropic contribution because no significant conformational changes of TMDs were observed in the binding process. In addition, the transporter binding step was mainly driven by hydrogen-bonds, π-π stacking, π-cation interactions and other weak electrostatic interactions (Xu et al., 2015). It is therefore suggested that the free energy along the access pathway is mainly governed by enthalpic contributions. However, tariquidar is highly flexible in the binding process. The umbrella sampling technique is an alternative method to obtain more accurate free energy curves. However, it heavily depends on the proper choice of reaction coordinates, which is a highly nontrivial task for systems as large and complex as P-gp.

## 5 Conclusion

**FIGURE 8:**
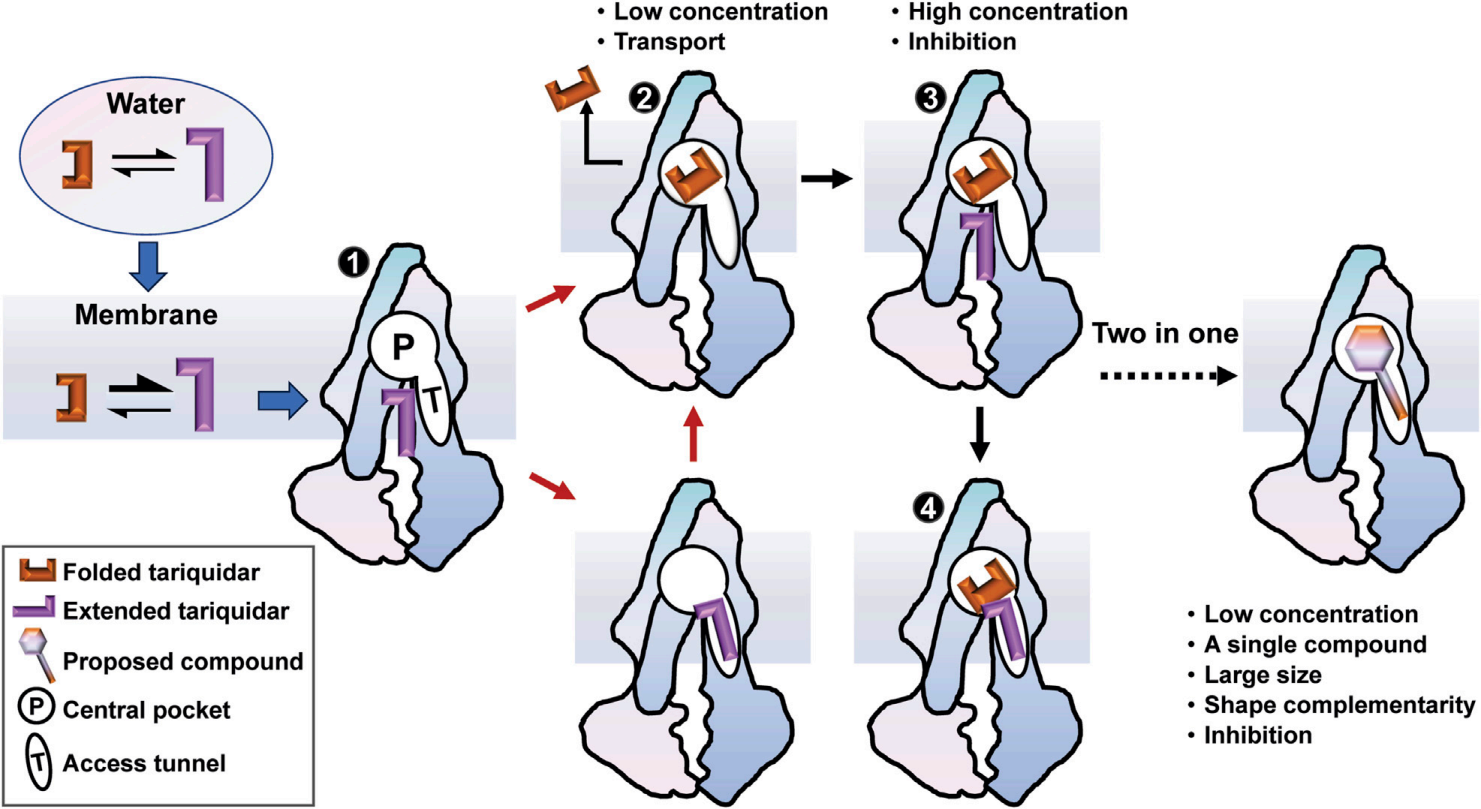
A membrane-assisted P-gp access and binding model for the third-generation inhibitors. The free tariquidar in aqueous solutions exists in a dynamic equilibrium between the extended and folded conformations, which shifts toward predominantly extended conformations in the lipid membrane. Dual asymmetric binding pathways from the cytoplasmic gate region to the central drug-binding pocket are initiated with an extended tariquidar conformation. At high concentrations, two inhibitor molecules simultaneously bind in the drug-binding pocket and the access tunnel, resulting in inhibitory effects by blocking the conformational changes required for substrate transport. At lower concentrations, the inhibitor only binds at the drug-binding pocket through the two binding pathways identified, leading to the subsequent transport. According to this model, we suggest a two-in-one strategy for the molecular design of more effective P-gp inhibitors.

In the present study, we used MD simulations to provide an atomic-level description of the conformational dynamics of human P-gp as well as the binding mechanism underlying tariquidar entry into the transmembrane sites. We propose a membrane-assisted binding mechanism in which the lipid membrane plays a pivotal role in pre-organizing tariquidar in an extended conformation that is not only preferable for the access and binding to both sites but also associated with the inhibitory activity (Figure 8). Notably, the significant structural adaptability of tariquidar highly contributes to the binding to the central pocket for the subsequent efflux rather than the regulatory site. Therefore, we further propose a two-in-one strategy for the design of P-gp inhibitors in which the large and flat compounds with reduced flexibility are able to modulate the transport function of P-gp at the level of a single molecule through simultaneously occupying the central drug-binding pocket and the access tunnel. In conclusion, our results not only suggest a novel binding mechanism for tariquidar, and potentially for the third-generation inhibitors, where the membrane lipids are seen to facilitate the access and binding to the transmembrane sites, but also have broader implications for molecular design of more potent and selective inhibitors targeting human P-gp for reversing drug resistance.

## Data Availability

The structural data used in this study can be downloaded from the RCSB Protein Data Bank (RCSB PDB, https://www.rcsb.org). Data supporting this study are included within the article and Supplementary Material. The trajectory data generated by MD simulations are available from the corresponding author on reasonable request.

## Abbreviations

P-gp, P-glycoprotein; ABC, ATP-binding cassette; BBB, blood-brain barrier; MDR, multidrug resistance; CNS, central nervous system; PNEB, partial nudged elastic band; MD, molecular dynamics; TMDs, transmembrane domains; NBDs, nucleotide-binding domains; POPC, 1-palmitoyl-2-oleoyl-sn-glycero-3-phosphocholine; MM/GBSA, Molecular Mechanics Generalized Born Surface Area; RMSD, root-mean-square deviations; Rgyr, radius of gyration; bRo5, beyond the rule of 5.
