# Supplementary material for: Membrane-assisted tariquidar access and binding mechanisms of human ATP-binding cassette transporter P-glycoprotein
Source: Front Mol Biosci. 2024 Mar 15;11:1364494. doi: 10.3389/fmolb.2024.1364494 (PMC10979361; doi:10.3389/fmolb.2024.1364494)
Supplement: Supplementary file 1 [file DataSheet1.docx]

Supplementary Material

**Table S1**. The PNEB simulation protocol for path optimization

| Step | Time scale (ps) | Spring constant (Kcal·mol^-1^) | Procedure | Start temperature (K) | End temperature (K) |
| --- | --- | --- | --- | --- | --- |
| 1 | 41 | 10 | linear heating | 0 | 310 |
| 2 | 200 | 50 | equilibrium | 310 | 310 |
| 3 | 50 | 50 | slow heating | 310 | 360 |
| 4 | 50 | 50 | equilibrium | 360 | 360 |
| 5 | 50 | 50 | slow heating | 360 | 410 |
| 6 | 50 | 50 | equilibrium | 410 | 410 |
| 7 | 50 | 50 | slow heating | 410 | 460 |
| 8 | 50 | 50 | equilibrium | 460 | 460 |
| 9 | 50 | 50 | slow heating | 460 | 510 |
| 10 | 50 | 50 | equilibrium | 510 | 510 |
| 11 | 50 | 50 | slow cooling | 510 | 460 |
| 12 | 50 | 50 | equilibrium | 460 | 460 |
| 13 | 50 | 50 | slow cooling | 460 | 410 |
| 14 | 50 | 50 | equilibrium | 410 | 410 |
| 15 | 50 | 50 | slow cooling | 410 | 360 |
| 16 | 50 | 50 | equilibrium | 360 | 360 |
| 17 | 50 | 50 | slow cooling | 360 | 310 |
| 18 | 50 | 50 | equilibrium | 310 | 310 |
| 19 | 50 | 50 | slow cooling | 310 | 260 |
| 20 | 50 | 50 | equilibrium | 260 | 260 |
| 21 | 50 | 50 | slow cooling | 260 | 210 |
| 22 | 50 | 50 | equilibrium | 210 | 210 |
| 23 | 50 | 50 | slow cooling | 210 | 160 |
| 24 | 50 | 50 | equilibrium | 160 | 160 |
| 25 | 50 | 50 | slow cooling | 160 | 110 |
| 26 | 50 | 50 | equilibrium | 110 | 110 |
| 27 | 50 | 50 | slow cooling | 110 | 60 |
| 28 | 50 | 50 | equilibrium | 60 | 60 |
| 29 | 50 | 50 | slow cooling | 60 | 0 |
| 30 | 50 | 50 | equilibrium | 0 | 0 |
| 31 | 400 | 50 | quenched MD | 0 | 0 |

**Table S2**. Docking scores of the retained 20 poses by LeDock.

| Pose no. | Score (kcal/mol) | Pose no. | Score (kcal/mol) |
| --- | --- | --- | --- |
| Pose 1 | -8.89 | Pose 11 | -7.91 |
| Pose 2 | -8.77 | Pose 12 | -7.71 |
| Pose 3 | -8.56 | Pose 13 | -7.70 |
| Pose 4 | -8.44 | Pose 14 | -7.57 |
| Pose 5 | -8.40 | Pose 15 | -7.53 |
| Pose 6 | -8.32 | Pose 16 | -7.48 |
| Pose 7 | -8.08 | Pose 17 | -7.43 |
| Pose 8 | -8.06 | Pose 18 | -7.36 |
| Pose 9 | -7.98 | Pose 19 | -7.31 |
| Pose 10 | -7.96 | Pose 20 | -7.09 |


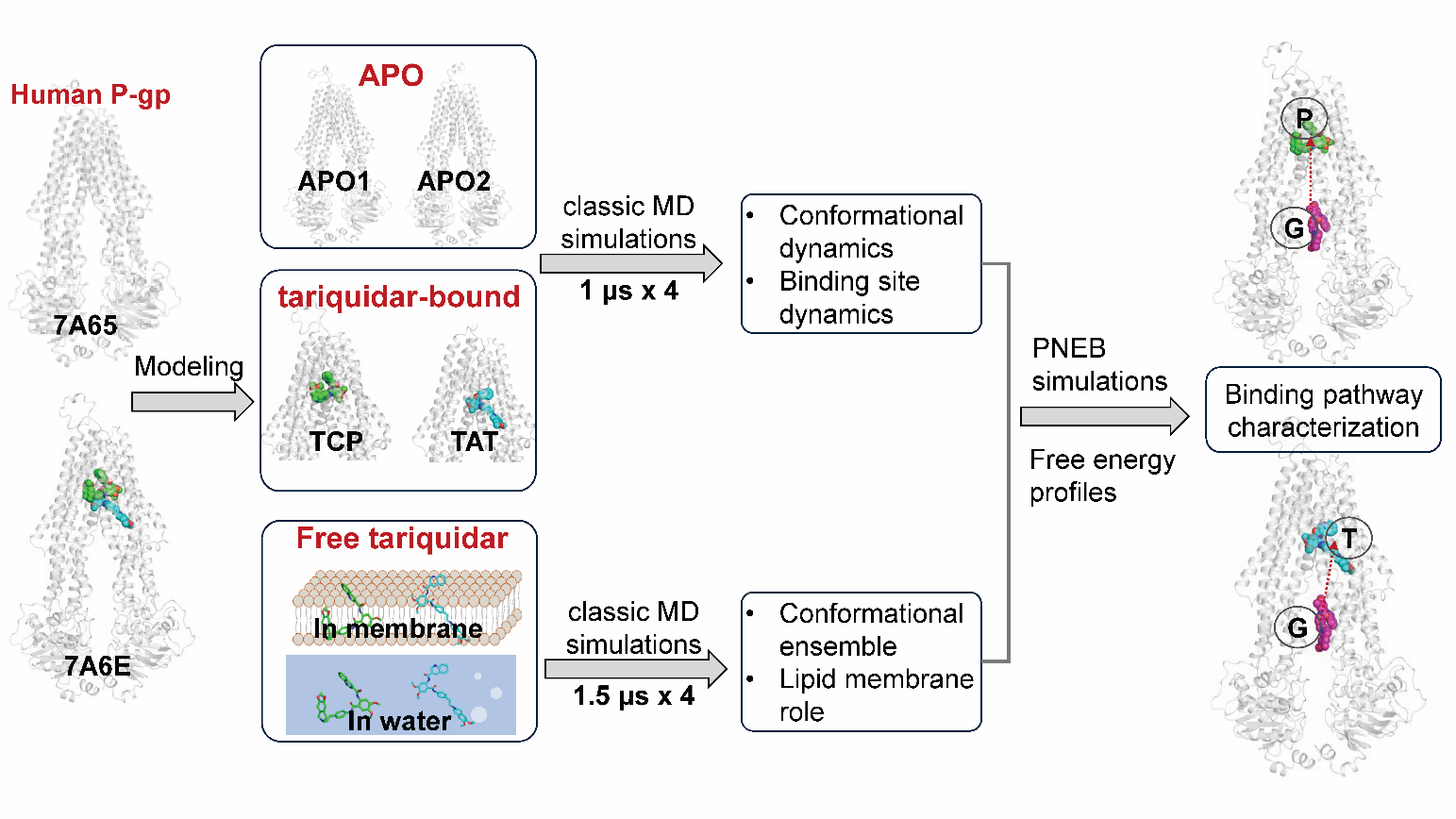


**Figure S1.** A schematic overview of the MD workflow in this study.


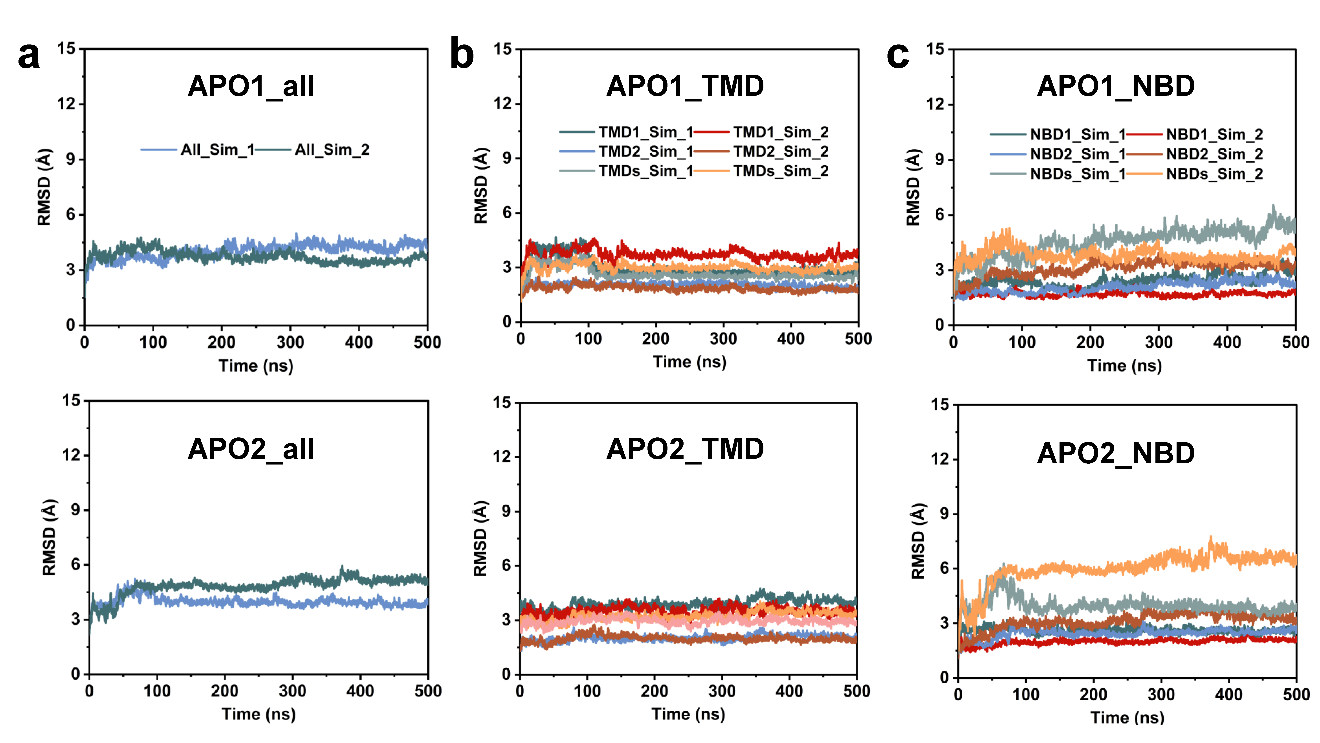


**Figure S2.** Conformational changes of drug-free (apo) human P-gp. (a) Time evolution of the overall root-mean-square deviations of C*α* (C*α*-RMSD) with respect to the starting structures of apo P-gp (PDB id: 7A65) across all MD simulations. (b and c) RMSD of individual domains, including the TMD and NBD monomers and dimers. Top panel: APO1 system; Bottom panel: APO2 system.


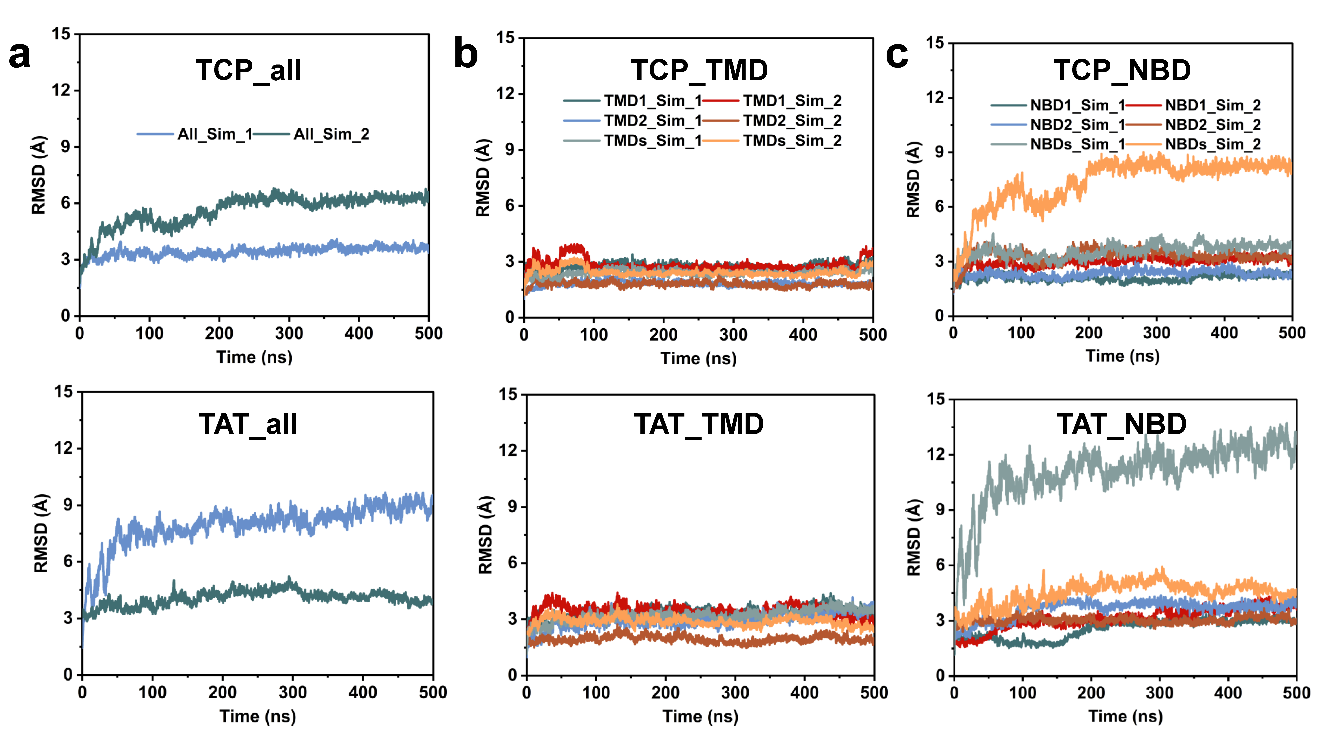


**Figure S3.** Conformational changes of singly tariquidar bound P-gp. (a) Time evolution of the overall C*α*-RMSD with respect to the starting structures of tariquidar-bound P-gp (PDB id: 7A6E) across all MD simulations. (b and c) C*α*-RMSD of individual domains, including the TMD and NBD monomers and dimers. Top panel: TCP system (tariquidar bound in the central pocket); Bottom panel: TAT system (tariquidar singly bound in the access tunnel).


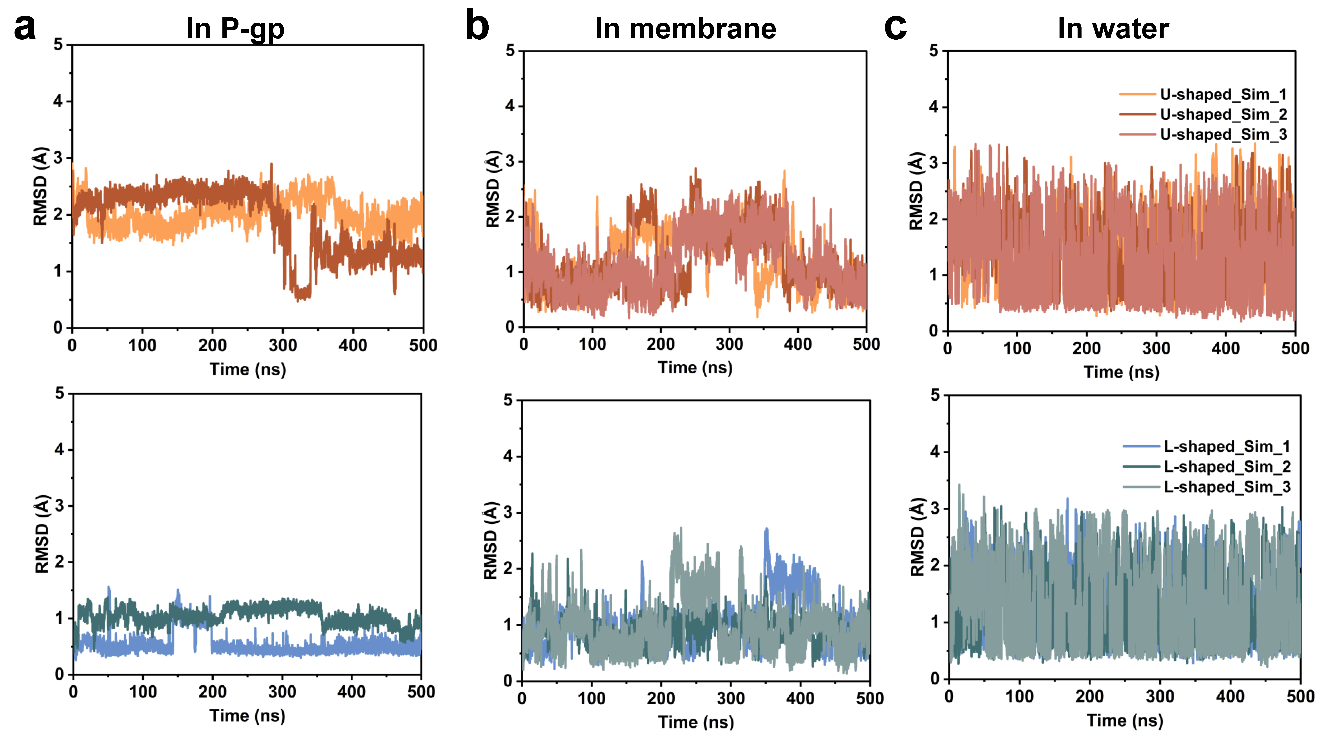


**Figure S4.** Conformational changes of tariquidar molecules in different environments. (a) Time evolution of C*α*-RMSD of tariquidar bound to the central pocket (upper panel) and the access tunnel (lower panel) in P-gp from the two unbiased MD simulations of TCP and TAT systems, respectively. (b) Time evolution of RMSD of tariquidar from three MD simulations in the POPC membrane. (c) Time evolution of RMSD of tariquidar from three MD simulations in water solution. MD simulations initiated by the folded TAR is shown in the upper panel, and those initiated by the extended TAR in the lower panel. The extended structure of tariquidar bound in the access tunnel was used as the reference structure for all RMSD calculations.


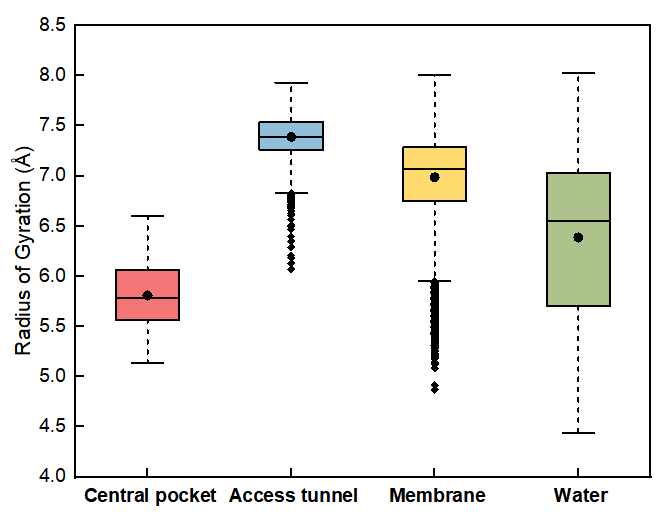


**Figure S5.** Boxplot of the radius of gyration (*R*_gyr_) for the conformational ensembles of tariquidar generated by MD simulations in transmembrane sites of human P-gp, lipid membrane and water solution.


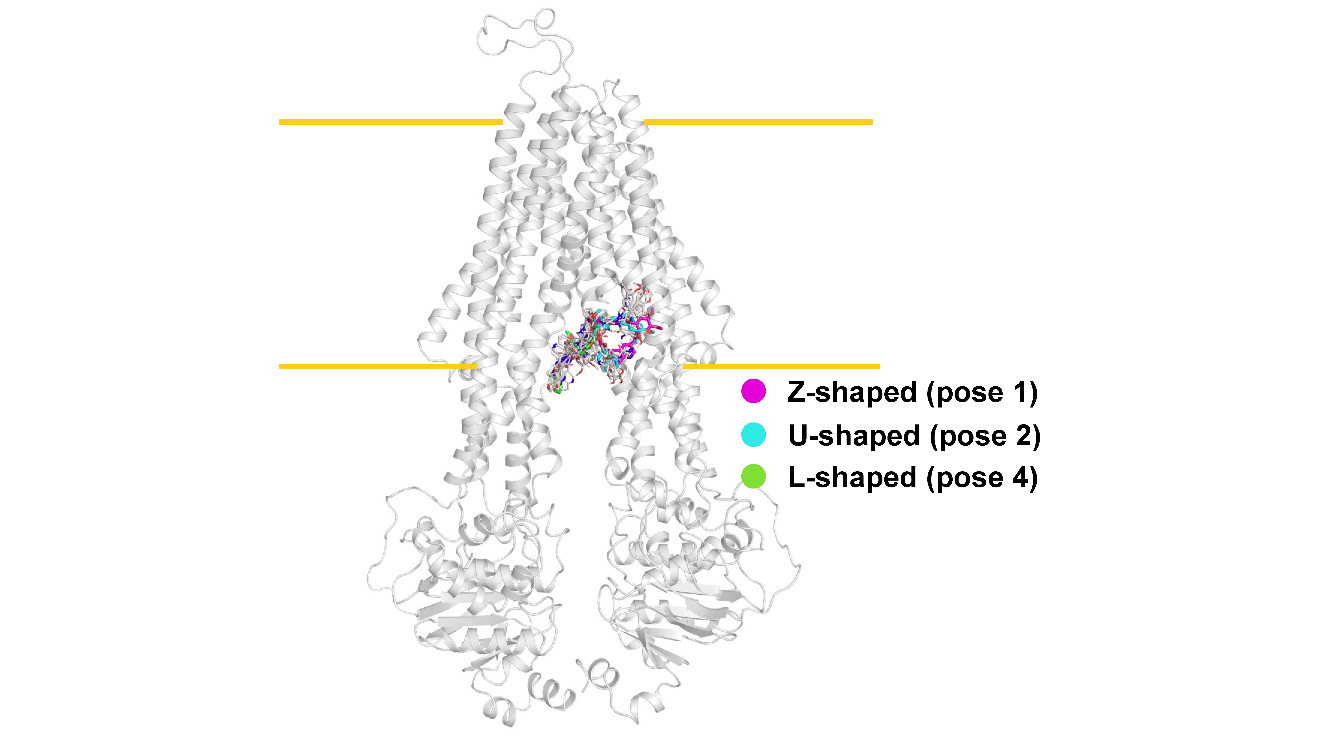


**Figure S6.** The 20 poses docked in the cytoplasmic gate region of human P-gp. The pose 3 is also a Z-shaped conformation, highly similar to the pose 1, and thus is excluded.


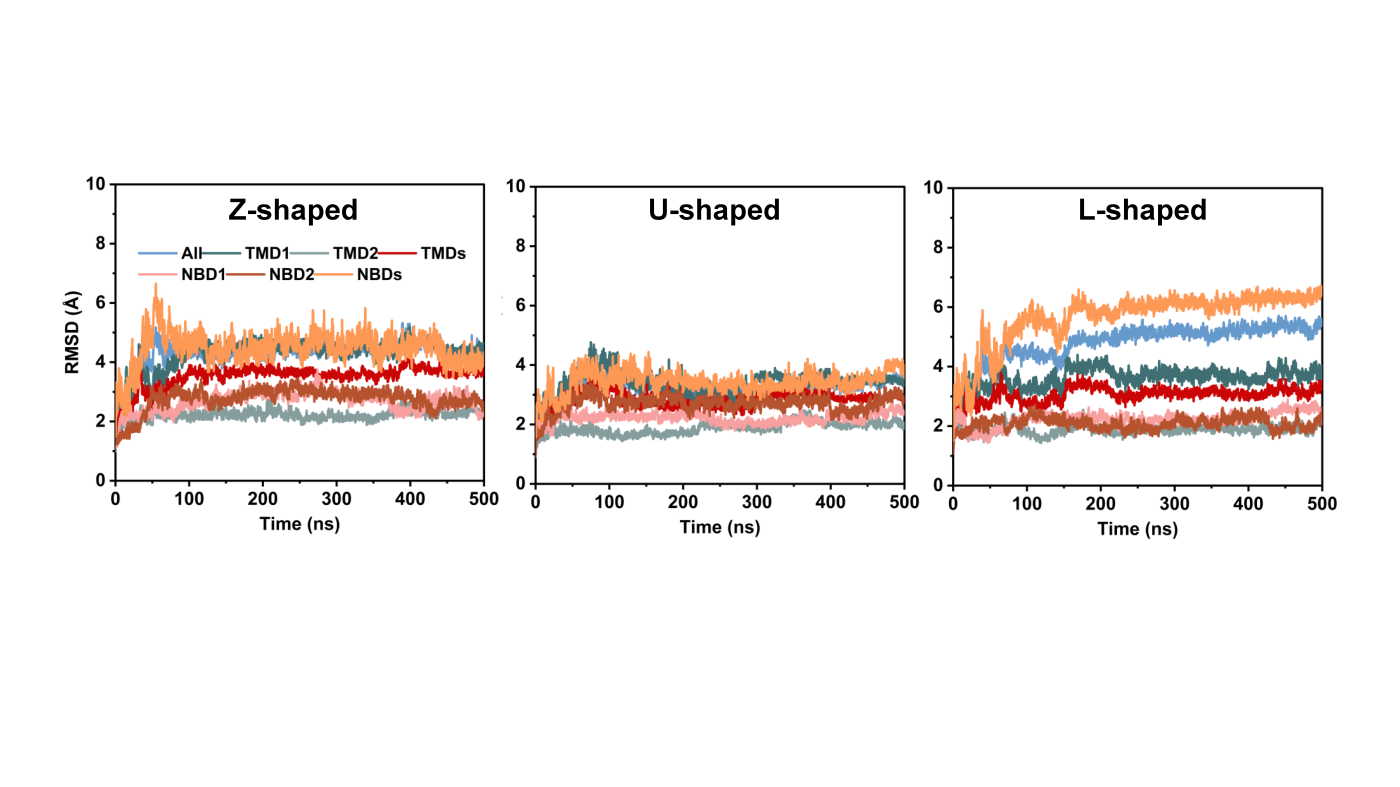


**Figure S7.** MD simulations of human P-gp with three tariquidar conformer bound in the cytoplasmic gate region generated by molecular docking. C*α*-RMSD calculations show that the transporter and the binding of tariquidar in the pre-bound state reach equilibrium quickly, and remain stable within the remaining 300 ns simulations. Therefore, the last snapshots of the three independent simulations were used as the starting point structures for PNEB calculation where tariquidar adopted L-, U- and Z-shaped conformations, respectively.


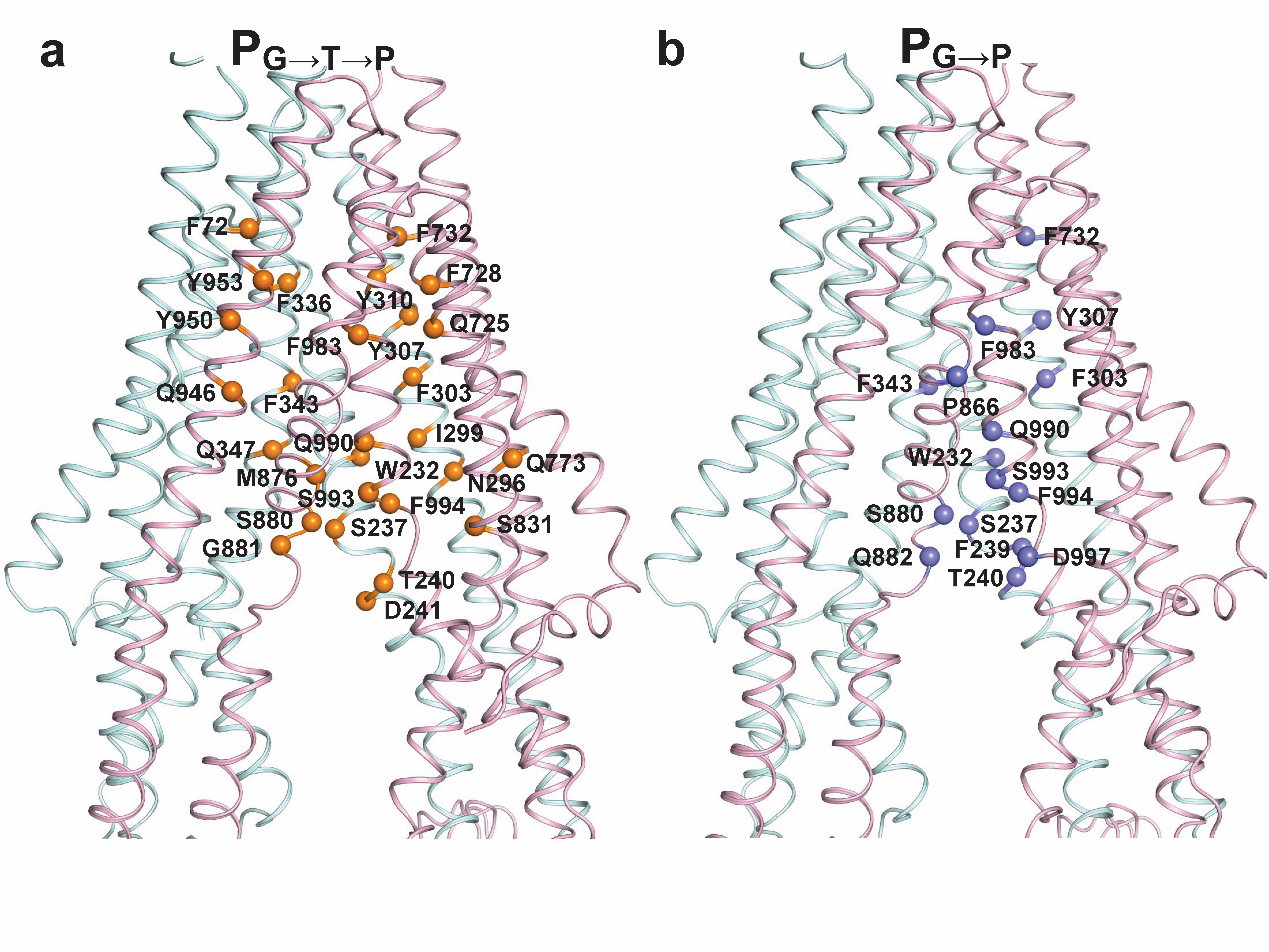


**Figure S8.** Close-up view of PNEB-optimized binding pathways in human P-gp. The pivotal tariquidar-interacting residues in the two pathways are labeled and shown as orange (a) and blue (b) balls, respectively.


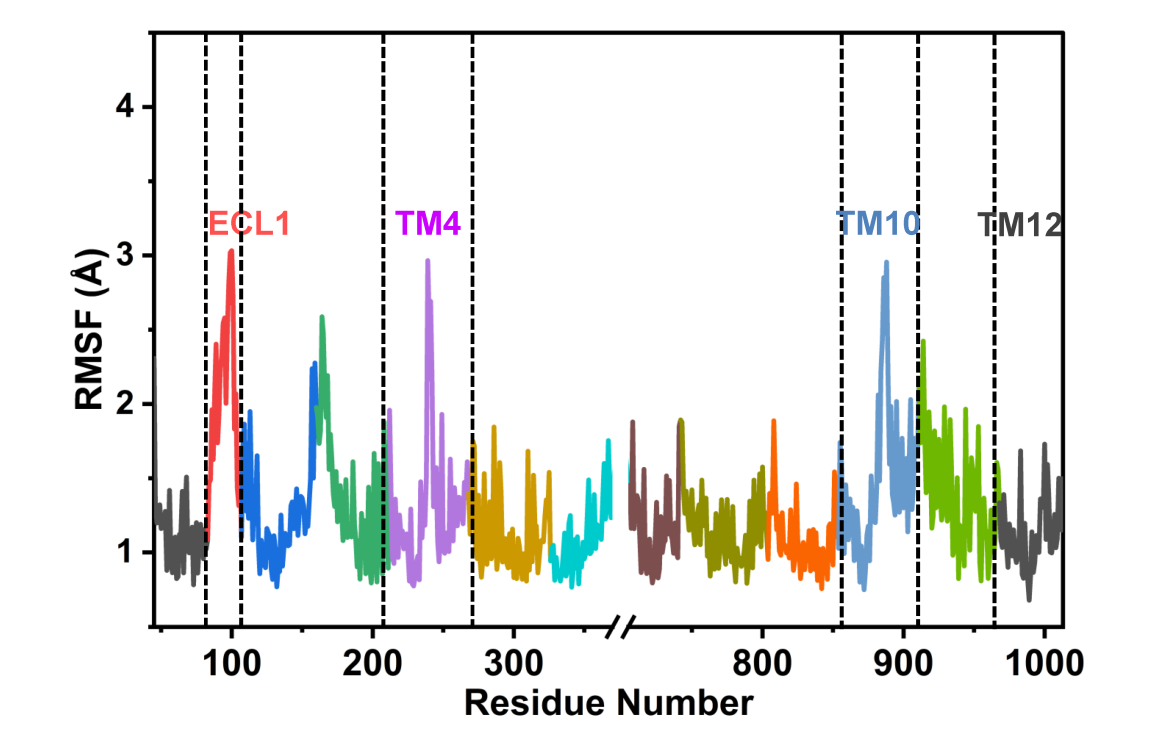


**Figure S9.** Root-mean-square fluctuations (RMSF) of C*α* in the TMDs of apo human P-gp. The average structure used as reference for RMSF calculation was derived from the last two 300 ns simulations of APO1 system. The 12 TMs are show in different colors. It can be observed that TM4 and TM10, whose flexibility is critical for drug binding and transport, fluctuate wildly in the apo state. The extracellular loop 1 (ECL1) connecting TM1 and TM2, which was re-modeled in this study, also shows a large fluctuation, consistent with the lower density in cryo-EM observation.
